# Insulin signaling in the long-lived reproductive caste of ants

**DOI:** 10.1126/science.abm8767

**Published:** 2022-09-01

**Authors:** Hua Yan, Comzit Opachaloemphan, Francisco Carmona-Aldana, Giacomo Mancini, Jakub Mlejnek, Nicolas Descostes, Bogdan Sieriebriennikov, Alexandra Leibholz, Xiaofan Zhou, Long Ding, Maria Traficante, Claude Desplan, Danny Reinberg

**Affiliations:** 1 Department of Biochemistry and Molecular Pharmacology, New York University School of Medicine, New York, NY 10016, USA.; 2 Howard Hughes Medical Institute, New York University School of Medicine, New York, NY 10016, USA.; 3 Department of Biology, Center for Smell and Taste, University of Florida, Gainesville, FL 32611, USA.; 4 Department of Biology, New York University, New York, NY 10003, USA.; 5 Guangdong Province Key Laboratory of Microbial Signals and Disease Control, South China Agricultural University, Guangzhou 510642, China.

## Abstract

In most organisms, reproduction is correlated with shorter life span. However, the reproductive queen in eusocial insects exhibits a much longer life span than that of workers. In *Harpegnathos* ants, when the queen dies, workers can undergo an adult caste switch to reproductive pseudo-queens (gamergates), exhibiting a five-times prolonged life span. To explore the relation between reproduction and longevity, we compared gene expression during caste switching. Insulin expression is increased in the gamergate brain that correlates with increased lipid synthesis and production of vitellogenin in the fat body, both transported to the egg. This results from activation of the mitogen-activated protein kinase (MAPK) branch of the insulin signaling pathway. By contrast, the production in the gamergate developing ovary of anti-insulin Imp-L2 leads to decreased signaling of the AKT/forkhead box O (FOXO) branch in the fat body, which is consistent with their extended longevity.

Differences in life span within a species offer an opportunity to investigate the regulatory processes involved in aging. Reproduction has an important influence on longevity: Genes that favor reproductive fitness normally shorten life span because animals allocate nutritional and metabolic resources for reproduction at the cost of longevity (1-3). Adaptive responses to dietary restriction in various species include reduced reproductive capability and prolonged life span (1, 2). The functional anticorrelation between reproduction and longevity involves the insulin/insulin-like growth factor (IGF) signaling pathway (IIS) because increased IIS activity required for reproduction leads to shorter life span in most animals (2, 4, 5).

Studies in *Caenorhabditis elegans, Drosophila*, mouse, and other model organisms have analyzed the effects of signaling pathways, particularly those of the IIS pathway, in regulating longevity (2, 3). In insects, the brain, fat body (the metabolic organ that is similar to the vertebrate liver and adipose tissue), and ovary are the primary tissues that regulate longevity and reproduction (3, 6). Ablation at the larval stages of the insulin-producing cells (IPCs) in the *Drosophila* brain causes lower female fecundity, increased storage of lipid, and extended life span (3, 7). The fat body mainly contains adipocytes and oenocytes (hepatocyte-like cells), which have essential roles in energy storage and utilization, pheromone synthesis, reproduction, and longevity (3, 6, 8). Up-regulation of the forkhead box O (FOXO) transcription factor, a negative effector of IIS, in the *Drosophila* fat body or adipose tissue in mice extends life span but reduces fecundity (2, 7). The ovary also has a role in the regulation of life span in some species: Removal of germline cells extends life span in *Drosophila* and *C. elegans* (7). However, in eusocial insects—such as ants, bees, wasps, and termites—the complete reproductive function of a colony depends on one or very few female individuals that have increased longevity [up to 30 times longer compared with their nonreproductive female nestmates (workers)] (9-11), despite sharing a similar genome. Although IIS has been widely studied in eusocial insects [for example, (12)], it is not clear yet how this reproduction-associated longevity is regulated at the cellular and molecular levels.

## Extended longevity upon caste switching from worker to pseudo-queen

**Fig. 1.**
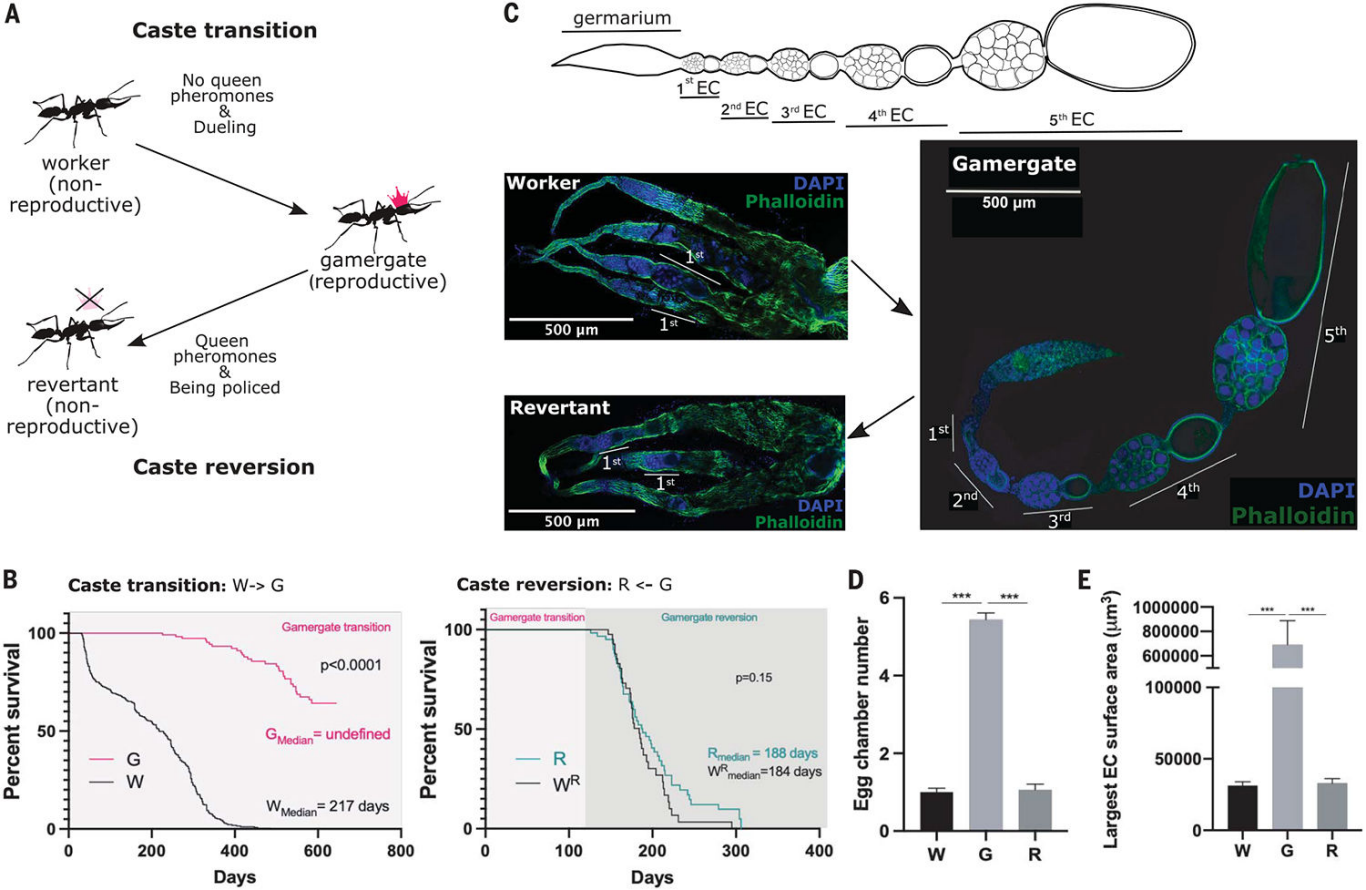
Phenotypic plasticity in the ant *Harpegnathos*. (**A**) *Harpegnathos* female workers retain reproductive potential, which is suppressed by the queen pheromone. Removal of the queen pheromone drives some nonreproductive workers to start dueling and become reproductive pseudo-queens, called gamergates (caste transition). The gamergates can transition anew to nonreproductive revertants in the presence of queen pheromones (policing and caste reversion). (**B**) Life span of ants during the caste transition and reversion. (Left) Survival curves of nonreproductive workers (W, black; *n* = 291) versus reproductive gamergates (G, pink; *n* = 40) during the W-to-G transition. (Right) Survival curves of revertants (R, green; *n* = 47) versus worker nestmates (W^R^, black; *n* = 36) during the G-to-R reversion. Gamergates derived from 3 months of transition (pink box) were subsequently subjected to reversion (green box). *P* values from Log-rank (Mantel-Cox) test are indicated in the plots. (**C**) Ovary development during the caste transition and reversion. (Top) Schematic of an ovariole within a gamergate ovary comprising a germarium and different stages of developing egg chambers (ECs). The sixth EC, which only contains a large oocyte without nurse cells, is not shown. (Bottom) Immunofluorescence (IF) staining of (top left) ovaries of worker and (bottom left) revertant and (right) a single ovariole of a gamergate with Phalloidin (green) and 4′,6-diamidino-2-phenylindole (DAPI) (blue). The numbers of developing ECs are indicated in the gamergate panel. (**D** and **E**) Quantifications of (D) the average number of developing ECs per ovariole and (E) the surface area of the largest EC in each ovariole of worker (W), gamergate (G), and revertant (R). *P* values are from Kruskal-Wallis test and multiple comparisons (***P < 0.001, *n* = 5 individuals). Bars and error bars represent mean ± SEM.

In the ant *Harpegnathos saltator* (*Hs*), when the queen in a colony dies or is removed, nonreproductive workers start dueling with each other. The winners gradually transition to becoming gamergates (pseudo-queens) that continue dueling, develop queen-like behavior, begin laying eggs, and exhibit a five-times life span extension (Fig. 1A) (10, 13, 14). Gamergates can also be reverted back to workers (“revertants”) when they are placed in a colony with an established reproductive caste, returning to a shortened life span (15). Although the median life span of regular workers (W) was 217 days (Fig. 1B and fig. S1A), the median life span of gamergates (G) was ~1100 days (10). The median life span of revertants (R) was 188 days, which was comparable with 184 days found in their worker nestmates (W^R^) (Fig. 1B and fig. S1A). This was shorter than the 217-day life span of regular workers, possibly because the harsh policing of gamergates by workers during the reversion induces colony stress.

To determine the reproductive potential of the castes, we inspected the ovaries of workers, gamergates, and revertants. Fertile gamergates had eight ovarioles (four on each side) that contained chains of four to six egg chambers, including some with fully developed oocytes (stage 6) (Fig. 1, C and D). By contrast, workers had ovaries with very small, partially developed ovarioles that each contained the germarium where stem cells are located, with zero to two early-stage egg chambers (stages 1 or 2) and no fully developed oocytes. Thus, ovary growth is blocked at the early stage of egg formation in workers but is activated in gamergates (Fig. 1, C and D). The ovaries of revertants also had zero to two immature egg chambers per ovariole (Fig. 1, C to E), which correlated with their loss of reproduction (15). We sought to address how life span could be increased during active reproduction in this species.

## Insulin expression is increased in the brain of the reproductive caste

**Fig. 2.**
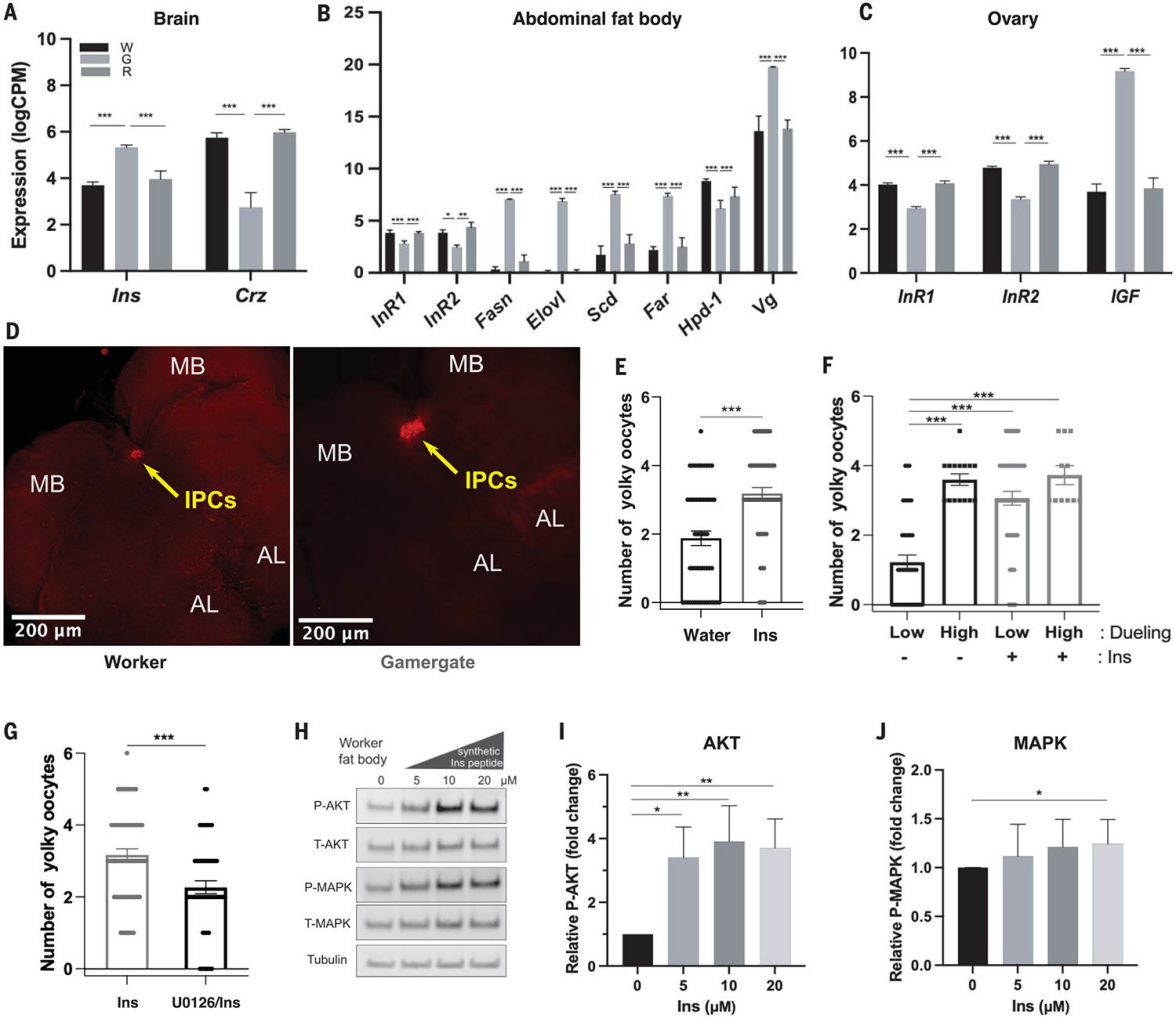
Ins-related gene expression in different castes and its induction of oogenesis through IIS-MAPK pathways. (**A** to **C**) RNA abundance of DEGs in the (A) central brain, (B) abdominal fat body, and (C) ovary in workers (W; black), gamergates (G; light gray), and revertants (R; dark gray). Data are from four biological replicates per caste. *P* values from EdgeR are indicated. *Hpd-1, 4-hydroxyphenylpyruvate dioxygenase*; *Fasn, fatty acid synthase*; *Elovl, fatty acid elongase*; *Scd, desaturase*; *Far, fatty acyl-CoA reductase*; *Vg, vitellogenin*; LogCPM, log counts per million. (**D**) Localization of (left) *Ins* mRNA in the worker and (right) gamergate brains by means of ISH. *Ins* mRNA is located in the IPCs (indicated with yellow arrows) located between mushroom bodies (MB). AL, antennal lobe. (**E** to **G**) Yolky oocyte production in (E) water- versus Ins-injected ants, (F) low- versus high-dueling ants, and (G) Ins-injected versus Ins and U0126 co-injected ants. In (E) and (G), ovary development is represented by the number of yolky oocytes per individual in all workers regardless of their dueling activity. Bars and error bars represent mean ± SEM (*P < 0.05, **P < 0.01, ***P < 0.001). (**H**) Western blot analysis of phosphorylated AKT (P-AKT), total AKT (T-AKT), phosphorylated MAPK (P-MAPK), total MAPK (T-MAPK), and tubulin levels in the worker fat body treated with different concentrations of synthetic Ins peptide (0, 5, 10, and 20 μM). (**I** and **J**) Quantification of fold changes in (I) relative P-AKT/T-AKT and (J) P-MAPK/T-MAPK. Relative levels of AKT and MAPK phosphorylation were normalized to the control fat body. *n* = 8 individuals; *P* values are from Kruskal-Wallis test with multiple comparisons. Bars and error bars represent mean ± SEM.

We performed bulk RNA sequencing from tissues that are important for reproduction and metabolism (brain, ovary, and fat body) from workers, gamergates, and revertants. The results confirmed the expression profile of previously characterized differentially expressed genes (DEGs), such as the gene encoding the neuropeptide Corazonin (Crz), which is highly expressed in the worker brain, and the egg yolk precursor *vitellogenin* (*Vg*) gene, which is increased in the gamergate fat body (Fig. 2, A and B, and table S1) (16). Additionally, we compared worker versus gamergate DEGs with gamergate versus revertant DEGs and found that the majority (>60%) of fat body and ovary genes with altered expression during the transition to gamergates returned to their worker expression in the revertants (fig. S1B). Several top Gene Ontology (GO) terms that were enriched among the DEGs up-regulated in the gamergate fat body were associated with fatty acid synthesis (fig. S1C and table S2). Fat body and ovary DEGs were also enriched in gamergates for terms associated with the IIS pathway, such as phosphatidylinositol 3-kinase (PI3K) signaling and insulin catabolic process (table S2). These results suggested that IIS-regulated metabolism is altered during the transition and reversion processes.

The IIS pathway has a central role in metabolism and, in particular, female reproduction: In most species studied, mutants in the IIS pathway exhibit extended life span and reduced reproduction (2, 3). We performed a phylogenetic analysis of genes related to insulin signaling of 71 Hymenopteran species (ants, bees, wasps, and sawflies) in the National Center for Biotechnology Information (NCBI) RefSeq database. In almost all species, including *Harpegnathos* but not in the ant *Camponotus floridanus*, there are two insulin-like peptides (ILPs): an insulin homolog (Ins) and an insulinlike growth factor homolog (IGF) (fig. S2). Ins contains A and B chains, whereas IGF has A and B and retains the C chain. Both ILPs can form three disulfide bonds, a structure that is essential for the interactions with their receptors (fig. S3, B and C).

We identified several genes differentially expressed between *Harpegnathos* gamergates and workers that were related to the IIS pathway: *Ins* mRNA was more abundant in the gamergate brain (ranking fifth among the gamergate-biased DEGs), whereas *IGF* mRNA was increased in the mature gamergate ovary (Fig. 2, A and C, and fig. S3A). By contrast, expression of the two genes encoding insulin receptors (InR1 and InR2) was decreased in the gamergate fat body and ovary (Fig. 2, B and C) but was unchanged in the brain. We generated mRNA probes and antibodies against *Hs*-Ins and *Hs*-IGF and examined their distribution by means of in situ hybridization (ISH) and immunofluorescence staining. As in *Drosophila*, the main source of Ins in the brain is the IPCs (Fig. 2D and fig. S4A) (17). *Ins* mRNA was more abundant in the cell body of IPCs in gamergates than in those of workers (Fig. 2D), whereas the Ins protein accumulated in both the cell body and the axons of the IPCs (fig. S4A). Increased *IGF* mRNA and protein were mainly detected in nurse cells and the follicle cells of the gamergate ovary (fig. S4B). This increase in ILPs in gamergates is consistent with the high metabolic requirement for egg production. However, high IIS activity should also lead to decreased life span.

## Altered metabolism in gamergates

Because IIS regulates metabolism and the gamergate-biased genes in the fat body were enriched in the GO terms associated with fatty acid synthesis, we analyzed metabolic changes in gamergates. The gamergate fat body exhibited increased expression of genes related to lipid metabolism and modifying enzymes—such as *fatty acid synthase* (*Fasn*), *fatty acid elongase* (*Elovl*), *desaturase* (*Scd*), and *fatty acyl-CoA reductase* (*Far*)—suggesting an active synthesis of lipids either for the production of the egg yolk or of cuticular hydrocarbons (CHCs), which are made up of long-chain hydrocarbons and constitute the queen pheromones (Fig. 2B) (14, 15, 18, 19). Thus, the fat body of gamergates exhibits transcriptional signatures of increased lipid production. The fat body of workers in the abdomen was large and white-colored, whereas it was reduced in size and had a yellowish color in the gamergate abdomen that was mainly occupied by the developed ovarioles (fig. S5A). We measured the lipid contents of the fat body [tri- and di-acylglycerol (TAG and DAG, respectively)] using an enzymatic conversion of glycerides into glycerol. Lipids were significantly decreased in the gamergate fat body compared with that of workers (fig. S5C), which is consistent with the observation that the dissected worker fat body floated in saline solution [1X phosphate-buffered saline (PBS)], whereas that of gamergate sank (fig. S5B). However, circulating lipids were increased in the hemolymph of gamergates, pointing to lower lipid storage and greater lipid mobilization in the reproductive gamergates (fig. S5, D and E). Moreover, Nile Red staining in the fat body revealed that lipid droplets were abundant in the enlarged adipocytes of workers but rarely detected in the smaller adipocytes of gamergates. Unlike in workers, lipids were mainly found in gamergate oenocytes (fig. S5, F to H). This result is consistent with the increased production of queen pheromone in gamergates because oenocytes are the main source for CHC pheromone synthesis in the abdomen (8, 18). We also measured carbohydrate abundance in dissected fat body tissue and in the hemolymph, including glycogen, trehalose, and glucose [supplementary materials (SM), materials and methods]. The abundance of carbohydrates, notably glycogen, was decreased in the fat body after the transition to gamergate, whereas the circulating trehalose and glucose in the hemolymph were unchanged (fig. S5, I and J).

## Insulin promotes oogenesis in workers but does not induce dueling

Ins and IGF activate ovary growth in multiple invertebrate and vertebrate species, and Ins induces reproduction in clonal raider ants (20). To test the role of IIS in *Harpegnathos* reproduction, we synthesized *Hs*-Ins. We injected the Ins peptide into the abdomen of ~2-week-old workers in queenless colonies (13, 14); 2 days after the setup of three independent colonies, half of the members (20 individuals) of each colony were injected with the Ins peptide, and the others were injected with water as a control. We scored the dueling behavior 5 days after dueling initiation and measured the development of the ovary. Ins injection did not induce dueling behavior in workers (27% of workers dueled in control colonies, whereas 19% of workers dueled in the Ins group; *n* = 3 colonies, *P* = 0.75) (fig. S6A). However, Ins-injected individuals developed ovarioles with an average of 3.2 egg chambers as compared with 1.9 in control ants (Fig. 2E). By contrast, an inactive form of Ins (B chain) (SM materials and methods) did not promote ovariole development (fig. S6B). Normally, in this experimental context, high dueling activity positively correlates with the probability of transitioning to a gamergate, and workers do not have developed ovaries. However, after injection of the Ins peptide, the number of yolky oocytes was increased by ~2.3 times in the workers that did not duel (3.0 versus 1.3; *P* < 0.001), but Ins injection in individuals that already dueled often had little further effect on ovarian development (3.8 versus 3.6) (Fig. 2F). Dueling and oocyte numbers were positively correlated in control workers [coefficient of determination (*R*^2^) = 0.54] but were not correlated in Ins-injected workers (*R*^2^ = 0.02) (fig. S6C). We also injected Ins into workers in colonies with established reproductive individuals, in which any worker that attempted to become a gamergate was subject to policing by other workers that detected its increased CHC profile (15, 18). Ins-injected ants were not policed while they developed ovarioles (Fig. S6D), suggesting that Ins is able to induce oogenesis but not dueling or production of queen pheromones. In clonal raider ants, Ins injections also promote egg production, although in a different context (20).

## Insulin can activate AKT and MAPK, but AKT is down-regulated in the gamergate fat body

We tested how Ins regulates the distinct downstream branches of the IIS pathway, IIS-AKT and IIS–MAPK (mitogen-activated protein kinase). ILPs can induce phosphorylation of the protein kinase AKT that prevents nuclear localization of the FOXO transcription factor. Thus, nuclear FOXO that promotes longevity is negatively regulated by IIS. ILPs can also induce phosphorylation of MAPK/ERK (extracellular signal–regulated kinase) to increase cell proliferation (21). We treated dissected fat bodies from workers with the synthetic *Hs*-Ins peptide. Ins activated phosphorylation of AKT (~3.5 times increase after Ins treatment; *P* < 0.01, *n* = 8 individuals) (Fig. 2, H and I), whereas the MAPK pathway was only mildly activated (~1.3 times increase after treatment; *P* < 0.05, *n* = 8 individuals) (Fig. 2, H and J).

**Fig. 3.**
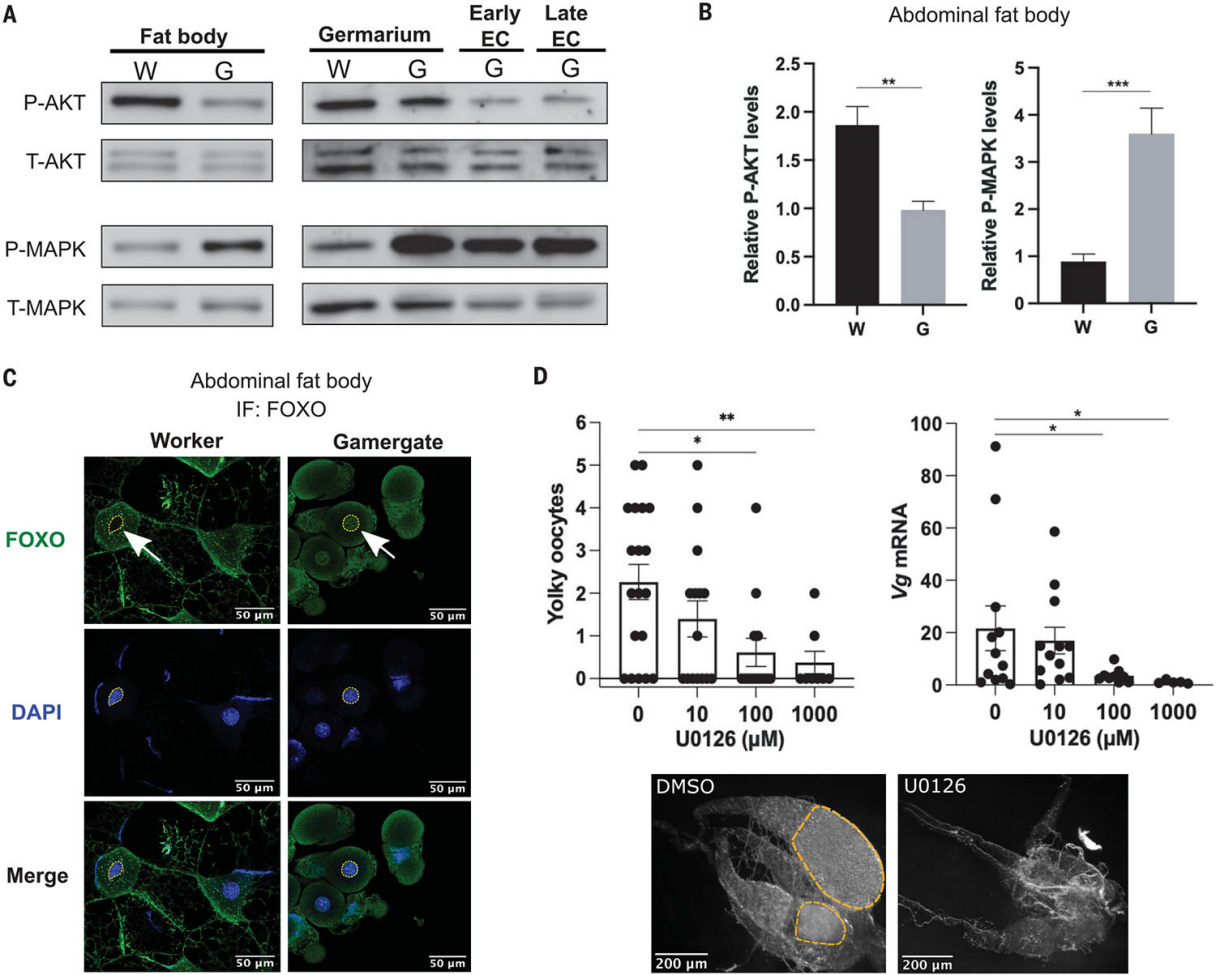
Activity of the IIS-AKT and IIS-MAPK pathways in different tissues and the requirement of MAPK activity for reproduction. (**A** to **C**) IIS activity is represented by phosphorylation of AKT and MAPK (P-AKT and P-MAPK, respectively), normalized by total AKT and MAPK (T-AKT and T-MAPK, respectively). (A) Western blot analysis of P-AKT, T-AKT, P-MAPK, and T-MAPK in (left) the fat body and (right) different parts of the dissected ovary [germarium, early egg chamber (EC), and late EC tissues from worker (W) versus gamergate (G)]. (B) Quantification of the relative P-AKT and P-MAPK levels in the fat body of W and G described in (A). *P* values are from unpaired Student’s *t* test (**P < 0.01, ***P < 0.001, *n* = 6 individuals). Bars and error bars represent mean ± SEM. (C) Immunofluorescence (IF) staining of transcription factor FOXO in the fat body as detected by *Hs*-FOXO antibody (green) and DNA staining with DAPI (blue). (Left) FOXO localization in the cytoplasm of worker fat body. (Right) FOXO localization in the nucleus of gamergate fat body. Nuclei were identified with DAPI and are indicated with arrows in the FOXO staining. (**D**) (Top) Averages of the yolky oocyte number per workers and quantitative RT-PCRs for *vitellogenin* (*Vg*) mRNA in abdominal fat body of workers 7 days after injection with either dimethyl sulfoxide (DMSO) or U0126 at different dosages (10, 100, and 1000 μM). (Bottom) Representative bright-field images of dissected ovaries. Data are from more than 10 biological replicates per condition. Bars and error bars indicate mean ± SEM. *P* values are from Kruskal-Wallis test with multiple comparisons. *Rpl32* is used as a reference gene for data normalization.

Because Ins leads to activation of both AKT and MAPK (Fig. 2, H to J, and fig. S3C), we expected to see globally increased activity of both IIS pathways in vivo in gamergates. To test this, we dissected multiple tissues, including the fat body and ovary, and analyzed the phosphorylation of MAPK. Consistent with the increased production of Ins in gamergates, MAPK phosphorylation was increased in the gamergate fat body (approximately four times increase; *n* = 6 individuals, *P* < 0.001) (Fig. 3, A and B) and in the ovary, including the germarium and the early- and late-stage egg chambers, and in the malpighian vesicle, the tissue equivalent to the vertebrate kidney (Fig. 3A and fig. S6E). However, MAPK phosphorylation was unchanged in the brain and the postpharyngeal gland (PPG), a tissue implicated in the synthesis and storage of cuticular CHC pheromones (fig. S6E) (22). Although MAPK can also be activated by the epidermal growth factor receptor tyrosine kinase (Egfr) (23), our transcriptome indicated that *Egfr* and its ligands, *Spitz* and *Vein* mRNAs, were less abundant in the gamergate fat body (fig. S3D), suggesting that the epidermal growth factor (EGF) pathway does not play a major role in activating MAPK in the fat body.

We also measured phosphorylation of AKT in multiple tissues in gamergates versus workers. Phosphorylation of AKT was significantly lower in gamergate fat bodies than in those of workers (approximately one half; *n* = 6 individuals, *P* < 0.01) (Fig. 3, A and B). This is consistent with evidence that reducing the AKT branch of insulin signaling in the fat body and adipose tissue lengthens the life span of other species (3). Phosphorylation of AKT was increased in the PPG of gamergates compared with that in workers but was at similar levels in the brain and the malpighian vesicle of workers and gamergates (fig. S6E) as well as in the germarium (Gm), the ovarian region that contains the germline stem cells in workers and gamergates (Fig. 3A). However, AKT phosphorylation was low in the ovary in early- and in late-stage egg chambers that are only present in gamergates (Fig. 3A). We analyzed the sub-cellular localization of FOXO in these tissues. If IIS-AKT was inactivated, unphosphorylated FOXO should be localized to the nucleus (24). FOXO was localized in the nucleus in the gamergate fat body, whereas it was localized in the cytoplasm of workers (Fig. 3C). mRNA for one of the target genes that are transcriptionally repressed by FOXO, *4-hydroxyphenylpyruvate dioxygenase* (*Hpd-1*) (25), was less abundant in the fat body of gamergates than in those of workers (Fig. 2B). *Hpd-1* encodes an enzyme that functions in the degradation of tyrosine, the precursor for biogenic amines, and dopamine is in greater abundance in gamergates than in workers (26). In the gamergate ovary, FOXO was localized in the nucleus in all follicle cells and in the nurse cells of egg chambers up to stage 4, whereas at stage 5, FOXO was localized to the cytoplasm in nurse cells (fig. S6F), suggesting that AKT activity is higher in late-stage nurse cells.

We conclude that increased Ins production in the gamergate brain leads to the activation of the MAPK pathway in the fat body and ovary. However, AKT activity is low in the fat body and part of the ovary of gamergates. How decreased AKT (but not MAPK) phosphorylation is achieved in gamergates is unclear.

## Pharmacological inhibition of MAPK activity affects ovary growth

Similar to what was observed in the clonal raider ants (20), Ins induces reproduction in *Harpegnathos* (Fig. 2, E and F). Because MAPK, but not AKT, appeared to be active in the gamergate fat body and ovary, we tested the effect of U0126 (a MAPK/ERK kinase inhibitor that prevents MAPK phosphorylation) on caste transition and ovary activation. U0126 inhibited MAPK phosphorylation in the dissected fat bodies of workers (to one-third of that of fat bodies from control ants) but only reduced AKT phosphorylation by 20%. In worker fat bodies exposed to both Ins and U0126, MAPK phosphorylation was reduced by one-half, and AKT phosphorylation was decreased by ~10% (fig. S6G).

We injected U0126 in workers during their transition to gamergates and measured *vitellogenin* expression as a molecular marker for egg production, then scored ovary growth 6 days after injection. U0126 treatment led to a concentration-dependent decreased expression of *vitellogenin* in the fat body as well as a decreased number of yolky oocytes in dueling individuals (Fig. 3D). Although oogenesis was promoted by Ins injection to workers (Fig. 2, E and F), U0126 impeded this effect (Fig. 2G). The germarium remained intact, suggesting that the inhibitor did not cause atrophy of the germline stem cell niche, which is present in both workers and gamergates.

We concluded that Ins from the brain can activate the MAPK pathway in the fat body and ovary and induces the production of mature egg chambers. MAPK activation seems to be necessary to promote *vitellogenin* expression in the fat body, ovary growth, and the transition to being reproductive. By contrast, AKT phosphorylation was decreased in the fat body and the developed ovaries of gamergates but remained comparable in the germarium of workers and gamergates. AKT activity in the germarium of both castes may function in germline stem cell maintenance and early differentiation, as it does in *Drosophila* (27). In this manner, workers can retain a functioning germ line that will allow them to reactivate oogenesis if they ever become gamergates.

## The IIS inhibitors Imp-L2 and ALS are up-regulated in the ovary, whereas InRs are decreased in the fat body

**Fig. 4.**
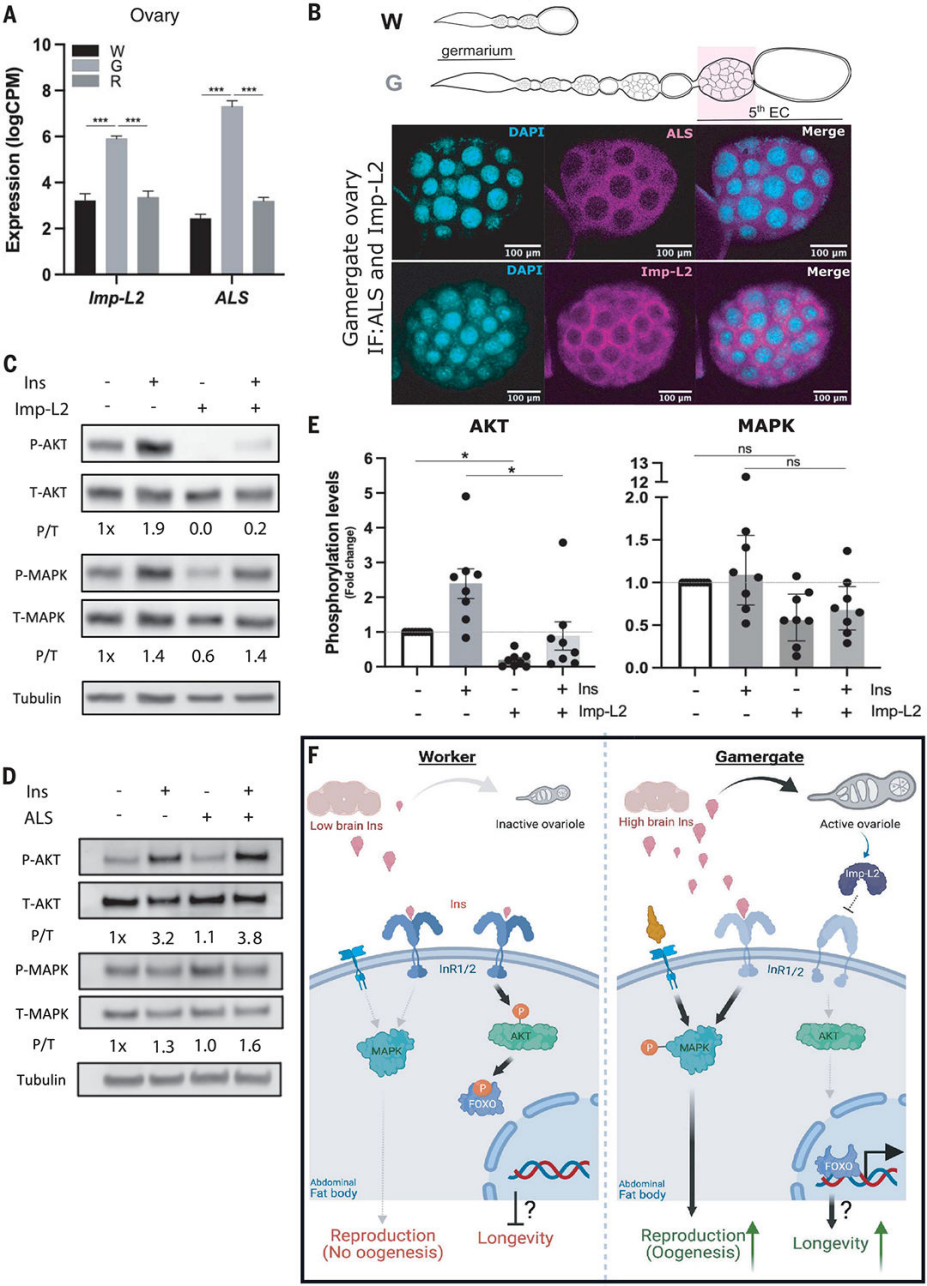
Imp-L2 produced from gamergate ovaries preferentially antagonizes the IIS-AKT pathway. (**A**) RNA abundance of ovarian *lmp-L2* and *ALS* in workers (W), gamergates (G), and revertants (R). Bars and error bars represent mean ± SEM. LogCPM, log counts per million. (**B**) IF staining of insulin/IGF-binding proteins (ALS and Imp-L2) in the late stages of the egg chambers of a gamergate ovary as indicated with a pink box in the schematic at top. Magenta indicates ALS and Imp-L2 at top and bottom, respectively. DAPI is shown in cyan. (**C** and **D**) Western blot analyses showing the effects of recombinant Imp-L2 or ALS (produced in Sf9 cells) on IIS-AKT and IIS-MAPK pathways in the abdominal fat body tissue dissected from workers. Levels of P-AKT, T-AKT, P-MAPK, T-MAPK, and tubulin detected in the fat body as a function of treatment with Ins peptide minus/plus recombinant (C) Imp-L2 or (D) ALS protein. (**E**) A quantitative plot of phosphorylation levels of AKT and MAPK from the worker fat body incubated with or without Imp-L2 in the presence and absence of Ins peptide. Bars and error bars indicate median with interquartile range. *P* values are from Mann-Whitney test (*P < 0.05, *n* = 8 individuals). (**F**) Proposed models of reproduction-associated longevity in ants. (Left) A short-lived worker. (Right) A long-lived gamergate. The basal level of insulin (Ins) secreted from the worker brain is sufficient to activate the IIS-AKT pathway through insulin receptors (InRs) in the abdominal fat body, resulting in FOXO phosphorylation and a normal life span in workers. By contrast, the high level of Ins in the gamergate brain promotes ovary maturation and contributes to MAPK activation in the fat body. An unidentified receptor (left of InR1/2) may also contribute to MAPK activation. Additionally, RNA abundance of *InR1/2* is decreased in the fat body of gamergate compared with that of worker (shown as lighter color), and the gamergate ovary produces Imp-L2 protein that antagonizes IIS-AKT signaling in the fat body. Together, this might lead to nuclear localization of FOXO and longevity in gamergates.

To explain the paradox of increased MAPK phosphorylation and decreased AKT phosphorylation in gamergates, we searched for candidate genes that could mediate the decreased IIS-AKT activity in the fat body and some ovarian tissues. In our differential expression analysis, *insulin receptor 1* and *2* (*InR1/2*) mRNAs were decreased in the gamergate fat body and whole ovary (Fig. 2B). However, this decrease was not sufficient to prevent MAPK phosphorylation in response to Ins treatment. Expression of two genes encoding secretory proteins that inhibit the IIS pathway was increased in the ovary of gamergates: *Imaginal morphogenesis protein-Late 2* (*Imp-L2*) and *Acid-labile Subunit* (*ALS*) (Fig. 4A). In *Drosophila*, Imp-L2 and ALS bind to circulating Dilp2 and Dilp5, the Ins homologs produced in brain IPCs. They are secreted into the hemolymph and antagonize IIS in peripheral tissues (28). Likewise, reduced expression of ILPs and increased expression of Imp-L2 appear to increase survival of mosquitoes (29). In mammals, ALS and IGF-binding protein 7 (IGFBP7), a homolog of Imp-L2, both bind plasma insulin and IGF in the circulation and subsequently restrain their interaction with their receptors (30, 31). IGFBP7 displays higher binding affinity to insulin than to IGF (30). Our expression analysis, paired with the evidence in insects and mammals, suggested that the elevated expression of *Imp-L2* and *ALS* genes in gamergate ovaries and their secretion into the hemolymph may antagonize insulin receptor activation in both mature ovarioles and the fat body.

The major sources of ALS in mammals are the liver and kidney (32), whereas *Imp-L2* mRNA is found in ovarian follicle cells (33). Quantitative reverse transcription polymerase chain reaction (RT-PCR) on multiple *Harpegnathos* tissues showed that *Imp-L2* and *ALS* mRNA were mainly expressed in ovaries, especially in late-stage egg chambers and yolky oocytes that were only present in gamergates but absent in workers. By contrast, their abundance was relatively low in other tissues, such as the brain, fat body, gut, and early-stage egg chambers (fig. S7A). We generated antibodies to Imp-L2 and ALS proteins. Proteins were only detected in late-stage egg chambers (Fig. 4B and fig. S7, B and C). Thus, Imp-L2 and ALS are predominantly expressed in gamergate ovaries, particularly in well-developed egg chambers from which they are secreted, and may act on abdominal tissues, including the fat body.

## Imp-L2 specifically blocks AKT in the fat body

*Imp-L2* and *ALS* expressed in the ovary may act as inhibitors that contribute to decreased AKT phosphorylation in the fat body and ovary of gamergates and thus increase life span. To test whether these proteins affect IIS in ants, we generated FLAG-tagged versions of *Hs-Imp-L2* and *Hs-ALS* in baculovirus and purified the proteins from lysates of transfected Sf9 insect cells using FLAG-based affinity purification (fig. S7D). We used purified proteins to treat dissected abdominal fat body tissues from workers. To minimize individual variations, the dissected fat body tissue from the same individual was separated for incubation with Ins, with or without Imp-L2 or ALS. Because of limited amounts of Imp-L2 purified from the baculovirus expression system, we used low doses of Ins (1 μM) to keep equal stoichiometry with Imp-L2 and ALS (1 μM). Although this dose of Ins significantly activated AKT, it was not sufficient to activate MAPK (Fig. 4, C to E).

Imp-L2 inhibited AKT phosphorylation: Even without Ins treatment, the baseline of AKT phosphorylation was strongly inhibited by Imp-L2 (to one-fifth of that of control tissue; *P* < 0.05, *n* = 8 individuals) (Fig. 4, C and E), whereas it did not significantly affect MAPK phosphorylation. Strong AKT phosphorylation after Ins treatment was completely suppressed by Imp-L2 (to one-third of that of control tissue; *P* < 0.05, *n* = 8 individuals) (Fig. 4, C to E), whereas MAPK phosphorylation was still not affected (Fig. 4, C and E), suggesting that Imp-L2 can specifically inhibit phosphorylation of AKT in gamergates. By contrast, ALS had no effect on AKT and MAPK phosphorylation (Fig. 4D). In support of our results, a study of human IGFBP7, a homolog of Imp-L2, was shown to inhibit IGF-induced phosphorylation of AKT in vitro but had less effect on MAPK phosphorylation, although this selectivity is not well understood [figure 1C in (34)].

Thus, we speculate that Imp-L2 produced by the ovary of gamergates acts as an Ins inhibitor to specifically reduce AKT activity in the fat body and ovary that might play a role in extending life span in gamergates. MAPK activity might be less affected and appears to be the primary regulator for initiating and sustaining the reproductive function of the ovary, in particular by producing vitellogenin and mobilizing lipids from the fat body that will accumulate in the egg. Therefore, phosphorylation of MAPK and inhibition of AKT phosphorylation in response to Ins could offer an effective solution to the discrepancy between increased insulin and reproduction and prolonged life span (Fig. 4F).

## Discussion

The traits that favor reproduction have particular importance in eusocial insects because the reproductive duty of the whole colony is placed on one or very few queens that are highly prolific. Such individuals are difficult or impossible to replace without disrupting the colony, so they must have a very long life span to allow the colony to thrive beyond the individual life of its workers (9, 35). Natural selection has thus resulted in increased life span in queens.

In *Harpegnathos*, production of Ins in the brain facilitates active reproduction through MAPK, but AKT phosphorylation in the fat body and developing ovary appears to be inhibited, which might contribute to extension of life span in gamergates. This local effect correlates with lower expression of InRs in the fat body and ovary and might result from the inhibitory effect of Imp-L2 secreted from the reproductive ovary.

The altered metabolism in the fat body of gamergates might lead to newly synthesized lipids to be directed through the hemolymph to the ovary to promote egg production. Because gamergates feed constantly, energy storage through lipid is less important. Although our work analyzed gamergates, *Harpegnathos* queens display even higher fecundity and longer life span than that of gamergates (35, 36), and higher expression of Ins has been found in queens in multiple ant species (20). Therefore, it is likely that our findings in gamergates apply to queens. In accordance, the queens of higher termites *Macrotermes natalensis* display higher Ins expression and lower fat storage compared with those of workers (37).

As in raider ants (20), the regulation of reproduction in *Harpegnathos* gamergates is achieved through increased insulin production. However, the peculiar lifestyle of raider ants (20) does not require an extended life span. To foster the extended life span of gamergates, the increased insulin in *Harpegnathos* does not stimulate the IIS-AKT pathway but instead only the IIS-MAPK branch. Our data demonstrate that Ins only mildly activates MAPK activation, although MAPK activity in the gamergate fat body is approximately four times greater than in workers; other ligands, in addition to Ins, may be responsible for MAPK activation in gamergates. We propose a model in which the transition of workers to gamergates is accompanied by the activation of IIS-MAPK, which might activate TOR (target of rapamycin) and its downstream transcription factor SREBP (sterol regulatory-element binding protein), a conserved pathway that might turn on expression of the *Fasn* and *vitellogenin* in the fat body and reactivates ovary growth (1). This is similar to what has been argued in *Drosophila*, in which vitellogenesis is regulated through the IIS-MAPK branch but not by IIS-AKT/FOXO (21, 38).

Reduced IIS-AKT activity in the fat body and early-stage egg chambers (stages 1 to 4) leads to the nuclear localization of FOXO and to reduced expression of a FOXO-negative target gene *Hpd-1* that functions in the degradation of tyrosine, the amino acid precursor for biogenic amines. In *C. elegans* and *Drosophila*, depletion of *Hpd-1* leads to extended life span, which was attributed to increased levels of dopamine and octopamine, which have a protective role in neuromuscular functions (39). Dopamine is greater in *Harpegnathos* gamergates than in workers (26). Thus, *Hpd-1* down-regulation might contribute to the extended longevity of reproductive gamergates.

Reduced IIS-AKT activity might be related to the production of Imp-L2 from the mature ovary, which is secreted into the hemolymph and might inhibit Ins-induced AKT phosphorylation in the fat body as observed ex vivo. This finding may offer a mechanism to slow down the aging process during active reproduction. By staying in the nest, queen ants are much better protected from extrinsic mortality for a longer life span (9, 40). However, intrinsic senescence that contributes to aging is also affected in *Harpegnathos* gamergates, and our findings could explain why higher fecundity promotes higher longevity: Imp-L2 is produced in the egg chambers of the mature ovary, and as more egg chambers are produced, this might further lower AKT signaling in the fat body, leading to a longer life span.

Our study in *Harpegnathos* gamergates reveals selectivity in the response of AKT and MAPK to insulin production. Activated MAPK and AKT differentially regulate metabolism, ovary growth, germline maintenance, and life span. Ants appear to restrict IIS hyperactivity throughout their very long reproductive life through selective inhibition of IIS-AKT possibly by Imp-L2 and through decreased expression of *InRs*, thus retarding aging and achieving longevity in the reproductive caste. This interplay, which evolved in ants and perhaps in other eusocial insects, may contribute to the unusual longevity associated with high reproduction observed in reproductive ants.

## Supplementary Material

science.abm8767_table_s1

science.abm8767_table_s2

science.abm8767_table_s3.xlsx

science.abm8767_table_s4.xlsx

science.abm8767_table_s5.xlsx

science.abm8767_sm_Sep12

science.abm8767_mdar_reproducibility_checklist.pdf
