# Supplementary material for: Insulin signaling in the long-lived reproductive caste of ants
Source: Science. Author manuscript; Available in PMC 2022 Oct 1. (PMC9526546; doi:10.1126/science.abm8767)
Supplement: science.abm8767_sm_Sep12 [file NIHMS1835898-supplement-science_abm8767_sm_Sep12.pdf]

Science AAAS

Supplementary Materials for

Insulin signaling in the long-lived reproductive caste of ants

Hua Yan *et al*.

Corresponding authors: Claude Desplan, cd38@nyu.edu; Danny Reinberg, danny.reinberg@nyulangone.org

Science **377**, 1092 (2022)
DOI: 10.1126/science.abm8767

The PDF file includes:

Materials and methods
Figs. S1 to S7
References

Other Supplementary Material for this manuscript includes the following:

MDAR Reproducibility Checklist
Tables S1 to S5

Materials and Methods

Regular maintenance of *Harpegnathos* ant colonies

Harpegnathos saltator colonies were initially transported from Jürgen Liebig's laboratory at Arizona State University. Ants were housed in plastic boxes (Pioneer Plastics, Inc.) with a plaster floor (Darby Dental, #8491560) inside a 12:12 h light/dark cycle and temperature-controlled room (22-25°C). Two compartments in a box divide the nest area for reproductive females, young workers (nurses) and the broods from the foraging arena for old workers (foragers). Ants and their broods were fed with pre-stung (paralyzed) crickets three times a week.

Caste transition (W to G) and reversion (G to R)

As shown in **Fig. S1A**., a queenless colony consisting of thirty of workers (~2-4 weeks post emergence) were setup for inducing a worker-to-gamergate transition for three months. Each individual was uniquely marked on thorax with Uni-paint markers. Well-known behavioral traits in gamergates: antennal dueling and egg laying events, were observed for identifying a mature gamergate (*14, 15, 26*). For the reversion, the mature gamergate (G), derived from 3 months of caste transition, was removed from the transition colony, individually isolated and constantly fed with small pre-stung crickets (1/4") for 4 weeks. Egg laying events were observed in the isolated mature gamergates. Workers (W) from the same transition colony were placed in another mature colony with established reproductive females to prevent their transition to gamergate after the gamergate removal and were used as a control worker in a lifespan experiment. The isolated gamergates were subsequently transferred into the mature colony where worker policing occurred. After two months, the reverted gamergates or revertants (R) fully exhibited worker-like behavior and stopped egg-laying (*15*). All tissues of workers, gamergates and revertants were harvested at the same chronological age (~6 months old). Worker and gamergate samples were harvested from the caste transition colony while revertants and their non-reproductive nestmates (W^R) were harvested independently from the reversion colony.

Identification of insulin-like peptides (ILPs) in *Harpegnathos saltator*

InterProScan was used to analyze all protein sequences of the 71 Hymenopteran genomes (Table S3), with annotations available in the NCBI RefSeq database. A total of 149 genes encoding proteins with "IPR016179", the characteristic domain of ILPs, were identified, among which all but four genes encode proteins longer than 100aa. Multiple sequence alignment of all protein sequences corresponding to the "IPR016179" domain region was created using the E-INS-i method of MAFFT v7.425 and filtered for gap-rich regions using TrimAl v1.4. A phylogenetic analysis was carried out using IQ-TREE v2.1.2 with 20 independent tree searches. The best-fitting evolutionary model ("JTT+G4") was selected automatically using ModelFinder, and branch supports were measured using the Ultrafast Bootstrap approach with 1000 replicates. The code is available at https:/github.com/bsierieb1/Ant_Insulin_Yan_et_al._2022

Transcriptome, GO enrichment analyses and RT-qPCR

Harpegnathos tissues were dissected from single individuals and homogenized in TriPure (Sigma). Three different tissues from 6-month-old workers (W), gamergates (G) and revertants (R) were collected including the central brain without optic lobes, whole ovary and abdominal fat body. Total RNA was purified by phenol/chloroform extraction. RNA was reverse transcribed to cDNA using QuantiTect Reverse Transcription Kit (Qiagen).

For library preparation, polyA+ RNA was isolated from total RNA using Dynabeads Oligo(dT) 25 (Thermo Fisher) beads. The 1st strand was synthesized by Superscript III and random hexamers (Life Technologies). The 2nd strand was synthesized with dUTP to generate strand asymmetry using DNA Pol I (NEB, M0209L) and E. coli DNA Ligase (Enzymatics, L6090L). RNA-seq libraries were constructed using the protocol in the lab. Sequencing was performed on a HiSeq 2500 (Illumina) at NYULH.

Reads were aligned with STAR (v.2.5.0b) on the *Harpegnathos* genome version 8.5. Read numbers in CPM (Counts Per Million) and differential expression analysis were calculated and performed using EdgeR. An adjusted p-value of less than 0.05 was considered significant**.** Gene identifiers, symbols and their closest homologs, which are available in the honeybee *Apis mellifera, Drosophila,* and mammals and obtained from the NCBI BLASTP web interface (http://blast.ncbi.nlm.nih.gov/Blast.cgi), flybase.org, alliancegenome.org and literatures, are reported in Table S4.

We predicted GO terms for HSAL51 transcripts (*41*) using CrowdGO (Table S2). CrowdGO was run with the CrowdGOFull pre-trained model. Biological Process terms predicted for transcripts were summarized by gene, and the resulting gene-to-GO mapping was used as input to topGO. Another input was a list of p values derived from differential expression analysis. Genes with false discovery rate-corrected p value less than 0.01 and the fold change sign corresponding to the selected direction of gene regulation (e.g. negative fold change for down-regulated genes, see below) were considered significantly differentially expressed. Gene enrichment analysis was run separately for each combination of the following factors: tissue (central brain, fat body, ovary), caste comparison (worker *vs.* gamergate, revertant *vs.* gamergate), and the direction of gene regulation (up-regulated in gamergate, down-regulated in gamergate). Gene enrichment was assessed using Fisher's exact test with elim algorithm, and GO terms with p value less than 0.01 were considered significantly enriched. For each combination of tissue and the direction of gene regulation, we intersected GO terms significantly enriched in both workers and revertants, sorted them by average ranked p value, selected top-ranked terms, and plotted the negative natural logarithm of the p values. The code is available at
https:/github.com/bsierieb1/Ant_Insulin_Yan_et_al._2022

For qPCR, mRNA expression levels were quantified using LightCycler® 480 SYBR Green I Master (Roche#04707516001) with a specific primer pair (See Table S5). *Ribosomal protein* (*Rpl32*) gene was used as normalization controls.

In situ hybridization (ISH) and immunofluorescence (IF) analyses

Tissue dissection was performed under 1x Phosphate Buffered Saline (PBS) supplemented with Protease and Phosphatase inhibitor cocktail (Thermo Scientific, #78443) on a clean, clear rubber pad using two fine forceps (Fine Science Tools, Dumont #5) and scissor (Fine Science Tools, #1500408). Dissected tissues were fixed using 4%

paraformaldehyde (PFA) (Electron Microscopy Sciences, #15710) diluted in 1x PBS with 0.1% Tween20 (0.1% PBST) at room temperature (RT) for 20 min on a shaker. Fixed tissues were washed three times with 0.1% PBST for 15 min each on a shaker.

ISH: Tissues were gradually dehydrated by incubating in serially diluted methanol at 25%, 50% and 90% at RT for 10 min each on a shaker. Tissues were rehydrated by performing a graded rehydration using 100%, 90%, 50%, and 25% methanol at RT for 10 min each. Tissue was then washed in 0.1% PBST twice for five min at RT while shaking. Post-fixation was performed by incubating in 4% PFA in 1x PBS for 20 min at RT on a shaker. Tissue was washed with PBST twice for five minutes while shaking. Tissue was permeabilized by incubating with Proteinase K (10 ug/ml) for 2 min at RT. Following incubation, the tissue was washed with 0.1% PBST for five min at RT while nutating. Another post fixation step was performed by incubating in 4% PFA in 1x PBS for 20 min at RT on a nutator. Tissue was subsequently washed with 0.1% PBST two times for 5 min at RT. The PBST was removed and replaced with pre-warmed hybridization buffer (50% formamide, 5x saline-sodium citrate (SSC), 5x Denhardt's solution, 250 ug/ml yeast tRNA, 500 ug/ml herring sperm DNA, 50 ug/ml Heparin, 2.5 mM EDTA, 0.1% Tween20, and 0.25% CHAPS) at 55° C for approximately two hours. Digoxigenin (DIG) labeled probe was synthesized using the DIG RNA labeling kit (SP6/T7) (Roche, #11175025910). Denatured DIG-labeled probe was hybridized at 55° C overnight. Hybridized tissue was then washed twice with warm hybridization solution for 40 min at 55° C and washed four times with 0.1% PBST for 10 min at RT. Detection of DIG-labeled probes was performed by incubating with anti-DIG antibody (1:2000, Roche #11093274910) in 0.1% PBST overnight at 4° C. Tissue was washed three times with 0.1% PBST and washed twice with AP buffer (100 mM NaCl, 50 mM $MgCl_2$, 100 mM Tris, pH 9.5, and 0.1% Tween20) for 10 min each at RT. Colorimetric detection was performed by incubating with NBT/BCIP solution (Roche, # 11697471001) for 10 min. The colorimetric reaction was halted by washing with 0.1% PBST two times for 5 min at RT while shaking.

IF: Fixed tissue was placed in custom antibodies of Ins, IGF, ALS, Imp-L2 and FOXO or pre-immune serum (1:100) in 0.1% PBSTx (TritonX-100) with 5% normal serum. Pre-immune serum was used as a negative control for IF staining. Tissue was incubated overnight at 4° C on a nutator. Subsequent removal of primary antibody was performed and tissue was washed four times for five min at RT in 0.1% PBSTx. Tissue was probed with anti-rabbit, -rat and –guinea pig secondary antibodies at RT for 1 hour on a nutator (1:500, ThermoFisher, #A21206, A21470 and A11073, respectively) and washed four times for five min at RT in 0.1% PBSTx. Counterstaining was performed by incubating with 2 μg/ml DAPI and 1:100 Alexa Fluor^{TM} 488 Phalloidin (ThermoFisher, #A12379) diluted in 0.1% PBSTx for 20 min at RT in the dark. Tissue was rinsed 5 times with 0.1% PBSTx, mounted in Focusclear and imaged using a Zeiss 880 Confocal microscope at NYULH.

Western analysis

Dissected tissues of foraging workers and mature gamergates were homogenized with lysis buffer (50 mM Tris-HCl, pH 7.8, 150 mM NaCl, and 1% Nonidet P-40) plus Halt protease/phosphatase inhibitor cocktail (78442). Approximately 10 μg of tissue lysate was loaded onto 8% Bis-Tris gels. PVDF membranes were used and blocked in TBST buffer (150 mM NaCl, 20 mM Tris-HCl, pH 7.5, and 0.05% v/v Tween20) containing 3% milk

(Carnation powdered milk) for 1 hour at RT and probed overnight at 4° C with primary rabbit antibodies (anti-phospho-AKT Ser505, #4054S, anti-phospho MAPK #9101S, two anti-total-AKT #9272S and #4691S, and anti-total-MAPK#4695S (Cell Signaling Technology, 1:2000), guinea pig anti-ImpL2 (1:1000) and rat anti-ALS (1:1000), diluted in TBST with 5% BSA and 0.04% sodium azide (NaN_3). Anti-rabbit (Promega, #W401B), anti-guinea pig (JacksonImmuno, #106035003) and anti-rat IgG HRP conjugate secondary antibodies (JacksonImmuno, #715035150) were used at a 1:5000 dilution in TBST containing 5% BSA or 3% milk. West FEMTO Maximum Sensitivity Substrate (Thermo Fisher, #34094) was used for signal detection. Membrane-bound antibodies were removed using Western Blot Stripping buffer (ThermoFisher, #21059). Band intensity was quantified using Fiji. Unpaired t test was performed using GraphPad Prism software.

Treatment of ant tissues by synthetic ILP peptides

Chemically synthetic Ins peptides were prepared by Phoenix Pharmaceuticals Inc. Two peptides (A and B chains) were synthesized and 3 disulfide bonds were formed.

A Chain: ARGIYEECCLNACTYNELSTYCGPQQ;
B Chain: QSDGSYALKWSMNVPQRYCGRRLSNALQTVCTGVYNNMF;
Disulfide bonds: [CysA9-CysB19], [CysA22-CysB31], [CysA8-CysA13].

Abdominal fat body tissues of workers were dissected in serum-free Schneider's medium (Invitrogen, #21720) supplemented with 1% streptomycin/penicillin. After 4 hours of starvation in serum-free medium, chemically synthetic Ins peptides were added to the media. After 30 min of treatment, media were discarded and protein lysates from tissues were subjected for Western blots. One-way ANOVA with Tukey's multiple comparisons test were performed using GraphPad Prism software.

Injections of Insulin peptide (Ins) and MAPK inhibitor (U0126)

For the *in vivo* experiment, 40 workers, two-weeks post-pupal emergence, were individually labeled with distinct paint dots and housed in a queenless condition. We injected approximately 1 µl of 100 µM synthetic insulin peptide into the worker abdomen (an opening between tergites) two days after setting up the colonies. Controls were injected with 1 µl water or 1 µl 100 µM Ins B chain as indicated. Ins B chain, without the A chain and disulfide bonds, is an inactive form of Ins. Antennal dueling behavior were observed for 5 days post-dueling initiation. All individuals were sacrificed after 5 days of the dueling tournament for ovary scoring by counting the number of yolky oocytes. For the U0126 experiment, workers were injected with approximately 1 µl of U0126 or 1% DMSO diluted with water. The ovary and abdominal fat body were collected six days post-injection for scoring ovary development and quantifying *vitellogenin* expression, respectively. Injections of both 100 µM U0126 and Ins peptide were performed separately. Half of the workers (20 workers) was first injected with either U0126 or 1% DMSO (control). Ins was subsequently injected in all workers (40 workers) on the following day. Dueling activities were monitored for 5 days and ovary development was scored 5 days post-dueling tournament. Three independent colonies were performed. Animals that did not survive the injections were excluded.

ALS and Imp-L2 protein production using baculovirus expression system

To purify ovarian ALS and ImpL-2 proteins, FLAG/HIS-tagged ALS and Imp-L2 were independently cloned into a baculovirus expression vector, pFASTBac1 (Invitrogen) and expressed in Sf9 cells (RRID: CVCL_0549). Infected Sf9 cells were resuspended in BC150 buffer (25 mM HEPES-NaOH, pH 7.5, 1 mM EDTA, 150 mM NaCl, 10% glycerol, 1 mM DTT, and 0.1% NP-40) with protease inhibitors (1 mM phenylmethlysulfonyl fluoride [PMSF], 0.1 mM benzamidine, 1.25 mg/mL leupeptin, and 0.625 mg/mL pepstatin A) and phosphatase inhibitors (20 mM NaF and 1 mM Na_3VO_4). Cells were lysed by sonication (Fisher Sonic Dismembrator model 100) and purified through FLAG-M2 agarose beads (Sigma).

Lipid and Carbohydrate Quantifications from the Tissues and Hemolymph

Lipid contents include multiple classes: triacylglycerol (TAG), phospholipid, steroid, glycolipid, etc. Some previous studies measured the lipid contents in the whole body (*42*), while others measured TAG and DAG (*37*), which represents fat storage and mobilization. We measured TAG/DAG specifically in the fat body and in the hemolymph.

Ants were rinsed with ultrapure water and anesthetized on ice. Extra liquid was removed. An abdomen and all legs were clipped off from the whole body. The abdomen obtained was dissected for the abdominal fat body and ovary under ice cold 1x PBS. The dissected tissues were homogenized in ice cold 1xPBS using an insulin needle (BD, #328291) or a pestle. The head and thorax were placed in a 0.5 ml tube with a small hole in the bottom inside a 1.5 ml collection tube. The hemolymph was collected after centrifugation at 3,000xg for 1 minute at 4° C. Approximately 1 µl of clear hemolymph was subjected to each lipid and sugar measurement. Quantification of TAG/DAG was performed using Infinity Triglycerides reagent (Thermofisher, #TR-22421), as described in the manufacturer's instructions.

For the measurements of trehalose and glucose, the lysates were diluted in the trehalose buffer (5 mM Tris pH 6.6, 137 mM NaCl, and 2.7 mM KCl). The samples obtained were incubated with porcine trehalase (Sigma, #T8778) at 37° C overnight. The measurements were performed using the Infinity Glucose Hexokinase reagent (Thermofisher, #TR-15421), as described in the manufacturer's instructions. To measure the glycogen level in the fat body, the dissected fat body was homogenized and incubated at 100°C for 10 min to inactivate enzymes. Quantification was performed using the Glycogen Assay Kit (ab65620) according to the manufacturer's instructions: glycogen was hydrolyzed to glucose using the enzyme glucoamylase. The oxidized product reacted with OxiRed probe. Fluorescence (Ex/Em= 535/587) was read in the FlexStation 3 Multi-mode Microplate Reader with the SoftMax Pro 7 software.

Quantification and Statistical Analysis

Statistical analysis of transcriptome data was performed using EdgeR. Other statistical analyses, including unpaired t test, one-way ANOVA with Tukey's multiple comparisons test, and Log-rank Mantel Cox test, as indicated in the Figure Legends and Methods, were performed using GraphPad Prism software. The value of n, mean ± SEM, and p value are reported in Results, Figures, and Figure Legends. Statistical significance is defined by $p<0.05$ (*), $p<0.01$ (**), or $p<0.001$ (***).

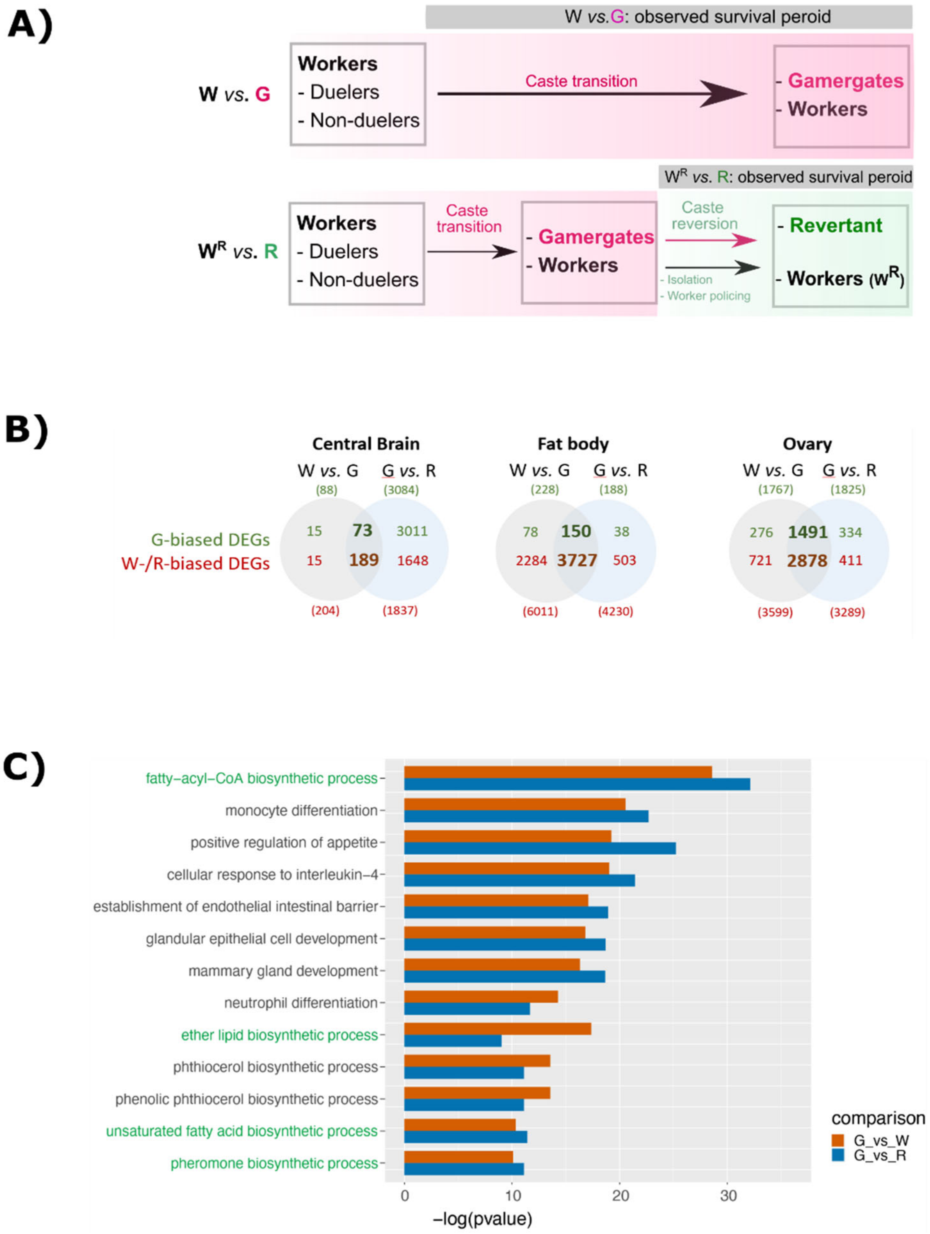
A)
W vs.G: observed survival peroid
W vs. G
Workers
- Duelers
- Non-duelers
Caste transition
- Gamergates
- Workers
W^R vs. R: observed survival peroid
W^R vs. R
Workers
- Duelers
- Non-duelers
Caste transition
- Gamergates
- Workers
Caste reversion
- Isolation
- Worker policing
- Revertant
- Workers (w^R)
B)
Central Brain
W vs. G
G vs. R
(88)
(3084)
15
73
3011
15
189
1648
(204)
(1837)
Fat body
W vs. G
G vs. R
(228)
(188)
78
150
38
2284
3727
503
(6011)
(4230)
Ovary
W vs. G
G vs. R
(1767)
(1825)
276
1491
334
721
2878
411
(3599)
(3289)
G-biased DEGs
W-/R-biased DEGs
C)
fatty-acyl-CoA biosynthetic process
monocyte differentiation
positive regulation of appetite
cellular response to interleukin-4
establishment of endothelial intestinal barrier
glandular epithelial cell development
mammary gland development
neutrophil differentiation
ether lipid biosynthetic process
phthiocerol biosynthetic process
phenolic phthiocerol biosynthetic process
unsaturated fatty acid biosynthetic process
pheromone biosynthetic process
0
10
20
30
-log(pvalue)
comparison
G_vs_W
G_vs_R

Fig. S1. Diagram of the timeline for caste transition [Worker (W) to Gamergate (G)] and reversion [Gamergate (G) to non-reproductive revertant (R)] and differentially expressed genes (DEGs) between W *vs.* G in the brain, abdominal fat body and ovary. (**A**) A colony consisting of 30 similarly aged matched workers initiates the dueling tournament, in which workers can be classified into two groups (dueling and non-dueling). The caste fate is determined based on dueling activity and oviposition. Survival rates of worker (W) and gamergate (G) are observed during the transition for over a year. For the process of G-to-R reversion, mature gamergates derived from the transition colonies were individually isolated in an empty nest followed by worker policing in other established gamergate colonies (revertant colonies). Their non-reproductive nestmates (workers) were directly transferred to the same revertant colonies, as a control for revertants (named W^R). The survival data of revertants (R) and non-reproductive nestmates (W^R) are collected during the reversion process. The survival data of W^R during the reversion serves as a baseline for the revertant's survival data. Thus, this does not represent the normal lifespan of a worker and shows a slight difference in lifespan compared to the workers in the W-to-G experiment. Individuals used in the reversion (lower image) are independent from the W-to-G transition (upper image). (**B**) Venn diagrams show the number of DEGs identified in three different tissues and the DEGs that overlap between the W *vs.* G. and G. *vs.* R. comparisons from each tissue. G-biased genes represent DEGs that are up-regulated in G compared to both W and R, while W- or R-biased genes are down-regulated DEGs in G compared to W or R, respectively. (**C**) A list of top biological process GO terms enriched in the overlapping G-biased genes in the fat body. GO terms are sorted by the negative natural logarithm of p values obtained from enrichment analysis of terms associated with DEGs in G *vs.* W and G *vs.* W comparisons (shown in orange and blue, respectively). G-biased GO terms related to lipid biosynthesis are highlighted in green.

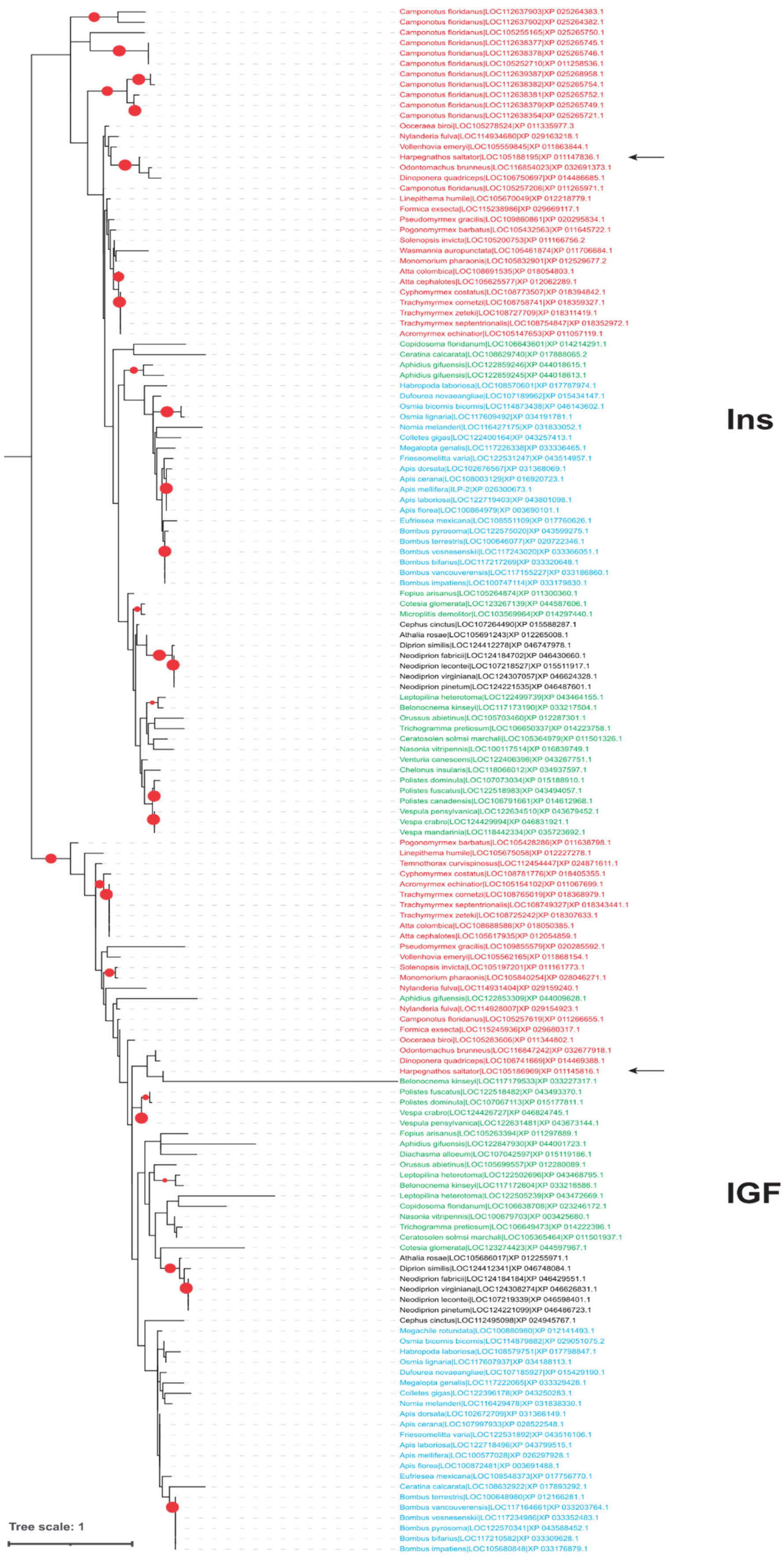
Camponotus floridanus|LOC112637903|XP 025264383.1
Camponotus floridanus|LOC112637902|XP 025264382.1
Camponotus floridanus|LOC105255165|XP 025265750.1
Camponotus floridanus|LOC112638377|XP 025265745.1
Camponotus floridanus|LOC112638378|XP 025265746.1
Camponotus floridanus|LOC105252710|XP 011258536.1
Camponotus floridanus|LOC112639387|XP 025268958.1
Camponotus floridanus|LOC112638382|XP 025265754.1
Camponotus floridanus|LOC112638381|XP 025265752.1
Camponotus floridanus|LOC112638379|XP 025265749.1
Camponotus floridanus|LOC112638354|XP 025265721.1
Ooceraea biroi|LOC105278524|XP 011335977.3
Nylanderia fulva|LOC114934680|XP 029163218.1
Vollenhovia emeryi|LOC105559845|XP 011863844.1
Harpegnathos saltator|LOC105188195|XP 011147836.1
Odontomachus brunneus|LOC116854023|XP 032691373.1
Dinoponera quadriceps|LOC106750697|XP 014486685.1
Camponotus floridanus|LOC105257206|XP 011265971.1
Linepithema humile|LOC105670049|XP 012218779.1
Formica exsecta|LOC115238986|XP 029669117.1
Pseudomyrmex gracilis|LOC109860861|XP 020295834.1
Pogonomyrmex barbatus|LOC105432563|XP 011645722.1
Solenopsis invicta|LOC105200753|XP 011166756.2
Wasmannia auropunctata|LOC105461874|XP 011706684.1
Monomorium pharaonis|LOC105832901|XP 012529677.2
Atta colombica|LOC108691535|XP 018054803.1
Atta cephalotes|LOC105625577|XP 012062289.1
Cyphomyrmex costatus|LOC108773507|XP 018394842.1
Trachymyrmex cornetzi|LOC108758741|XP 018359327.1
Trachymyrmex zeteki|LOC108727709|XP 018311419.1
Trachymyrmex septentrionalis|LOC108754847|XP 018352972.1
Acromyrmex echinatior|LOC105147653|XP 011057119.1
Copidosoma floridanum|LOC106643601|XP 014214291.1
Ceratina calcarata|LOC108629740|XP 017888065.2
Aphidius gifuensis|LOC122859246|XP 044018615.1
Aphidius gifuensis|LOC122859245|XP 044018613.1
Habropoda laboriosa|LOC108570601|XP 017787974.1
Dufourea novaeangliae|LOC107189962|XP 015434147.1
Osmia bicornis bicornis|LOC114873438|XP 046143602.1
Osmia lignaria|LOC117609492|XP 034191781.1
Nomia melanderi|LOC116427175|XP 031833052.1
Colletes gigas|LOC122400164|XP 043257413.1
Megalopta genalis|LOC117226338|XP 033336465.1
Frieseomelitta varia|LOC122531247|XP 043514957.1
Apis dorsata|LOC102676567|XP 031368069.1
Apis cerana|LOC108003129|XP 016920723.1
Apis mellifera|ILP-2|XP 026300673.1
Apis laboriosa|LOC122719403|XP 043801098.1
Apis florea|LOC100864979|XP 003690101.1
Eufriesea mexicana|LOC108551109|XP 017760626.1
Bombus pyrosoma|LOC122575020|XP 043599275.1
Bombus terrestris|LOC100646077|XP 020722346.1
Bombus vosnesenskii|LOC117243020|XP 033366051.1
Bombus bifarius|LOC117217269|XP 033320648.1
Bombus vancouverensis|LOC117155227|XP 033186860.1
Bombus impatiens|LOC100747114|XP 033179830.1
Fopius arisanus|LOC105264874|XP 011300360.1
Cotesia glomerata|LOC123267139|XP 044587606.1
Microplitis demolitor|LOC103569964|XP 014297440.1
Cephus cinctus|LOC107264490|XP 015588287.1
Athalia rosae|LOC105691243|XP 012265008.1
Diprion similis|LOC124412278|XP 046747978.1
Neodiprion fabricii|LOC124184702|XP 046430660.1
Neodiprion lecontei|LOC107218527|XP 015511917.1
Neodiprion virginiana|LOC124307057|XP 046624328.1
Neodiprion pinetum|LOC124221535|XP 046487601.1
Leptopilina heterotoma|LOC122499739|XP 043464155.1
Belonocnema kinseyi|LOC117173190|XP 033217504.1
Orussus abietinus|LOC105703460|XP 012287301.1
Trichogramma pretiosum|LOC106650337|XP 014223758.1
Ceratosolen solmsi marchali|LOC105364979|XP 011501326.1
Nasonia vitripennis|LOC100117514|XP 016839749.1
Venturia canescens|LOC122406396|XP 043267751.1
Chelonus insularis|LOC118066012|XP 034937597.1
Polistes dominula|LOC107073034|XP 015188910.1
Polistes fuscatus|LOC122518983|XP 043494057.1
Polistes canadensis|LOC106791661|XP 014612968.1
Vespula pensylvanica|LOC122634510|XP 043679452.1
Vespa crabro|LOC124429994|XP 046831921.1
Vespa mandarinia|LOC118442334|XP 035723692.1
Pogonomyrmex barbatus|LOC105428286|XP 011638798.1
Linepithema humile|LOC105675058|XP 012227278.1
Temnothorax curvispinosus|LOC112454447|XP 024871611.1
Cyphomyrmex costatus|LOC108781776|XP 018405355.1
Acromyrmex echinatior|LOC105154102|XP 011067699.1
Trachymyrmex cornetzi|LOC108765019|XP 018368979.1
Trachymyrmex septentrionalis|LOC108749327|XP 018343441.1
Trachymyrmex zeteki|LOC108725242|XP 018307633.1
Atta colombica|LOC108688586|XP 018050385.1
Atta cephalotes|LOC105617935|XP 012054859.1
Pseudomyrmex gracilis|LOC109855579|XP 020285592.1
Vollenhovia emeryi|LOC105562165|XP 011868154.1
Solenopsis invicta|LOC105197201|XP 011161773.1
Monomorium pharaonis|LOC105840254|XP 028046271.1
Nylanderia fulva|LOC114931404|XP 029159240.1
Aphidius gifuensis|LOC122853309|XP 044009628.1
Nylanderia fulva|LOC114928007|XP 029154923.1
Camponotus floridanus|LOC105257619|XP 011266655.1
Formica exsecta|LOC115245936|XP 029680317.1
Ooceraea biroi|LOC105283606|XP 011344802.1
Odontomachus brunneus|LOC116847242|XP 032677918.1
Dinoponera quadriceps|LOC106741669|XP 014469388.1
Harpegnathos saltator|LOC105186969|XP 011145816.1
Belonocnema kinseyi|LOC117179533|XP 033227317.1
Polistes fuscatus|LOC122518482|XP 043493370.1
Polistes dominula|LOC107067113|XP 015177811.1
Vespa crabro|LOC124426727|XP 046824745.1
Vespula pensylvanica|LOC122631481|XP 043673144.1
Fopius arisanus|LOC105263394|XP 011297889.1
Aphidius gifuensis|LOC122847930|XP 044001723.1
Diachasma alloeum|LOC107042597|XP 015119186.1
Orussus abietinus|LOC105699557|XP 012280089.1
Leptopilina heterotoma|LOC122502696|XP 043468795.1
Belonocnema kinseyi|LOC117172604|XP 033216586.1
Leptopilina heterotoma|LOC122505239|XP 043472669.1
Copidosoma floridanum|LOC106638708|XP 023246172.1
Nasonia vitripennis|LOC100679703|XP 003425680.1
Trichogramma pretiosum|LOC106649473|XP 014222396.1
Ceratosolen solmsi marchali|LOC105365464|XP 011501937.1
Cotesia glomerata|LOC123274423|XP 044597967.1
Athalia rosae|LOC105686017|XP 012255971.1
Diprion similis|LOC124412341|XP 046748084.1
Neodiprion fabricii|LOC124184184|XP 046429551.1
Neodiprion virginiana|LOC124308274|XP 046626831.1
Neodiprion lecontei|LOC107219339|XP 046598401.1
Neodiprion pinetum|LOC124221099|XP 046486723.1
Cephus cinctus|LOC112495098|XP 024945767.1
Megachile rotundata|LOC100880980|XP 012141493.1
Osmia bicornis bicornis|LOC114879882|XP 029051075.2
Habropoda laboriosa|LOC108579751|XP 017798847.1
Osmia lignaria|LOC117607937|XP 034188113.1
Dufourea novaeangliae|LOC107185927|XP 015429190.1
Megalopta genalis|LOC117222065|XP 033329428.1
Colletes gigas|LOC122396178|XP 043250283.1
Nomia melanderi|LOC116429478|XP 031838330.1
Apis dorsata|LOC102672709|XP 031366149.1
Apis cerana|LOC107997933|XP 028522548.1
Frieseomelitta varia|LOC122531892|XP 043516106.1
Apis laboriosa|LOC122718496|XP 043799515.1
Apis mellifera|LOC100577028|XP 026297928.1
Apis florea|LOC100872481|XP 003691488.1
Eufriesea mexicana|LOC108548373|XP 017756770.1
Ceratina calcarata|LOC108632922|XP 017893292.1
Bombus terrestris|LOC100648980|XP 012166281.1
Bombus vancouverensis|LOC117164661|XP 033203764.1
Bombus vosnesenskii|LOC117234986|XP 033352483.1
Bombus pyrosoma|LOC122570341|XP 043588452.1
Bombus bifarius|LOC117210582|XP 033309628.1
Bombus impatiens|LOC105680848|XP 033176879.1
Ins
IGF
Tree scale: 1

Fig. S2. **Phylogenetic tree of *Ins* and *IGF* genes, separated into two groups**. Genes from ants, bees, wasps, and sawflies are indicated in red, blue, green, and black font respectively. Most hymenopteran species analyzed in this study, including *Harpegnathos saltator* indicated by arrows, clearly contain two *ILPs*: *Ins* and *IGF*. Numbers on internal branches represent support values as measured by the Ultrafast Bootstrap approach with 1000 replicates. Red dots in the tree indicate bootstrap support >= 95%.

A)

Rank	Characterized gene ID	logFC	logCPM	PValue	FDR	Annotation
1	LOC105185161	-9.0327	7.415663	7.67E-26	4.85E-22	arylphorin subunit alpha
2	LOC105181726	-4.8595	14.07645	1.85E-15	2.60E-12	vitellogenin-like
3	LOC105188568	-1.0116	10.60769	1.92E-12	1.43E-09	centrosomal protein of 78 kDa
4	LOC112589399	-4.2257	5.043575	6.43E-12	4.52E-09	chymotrypsin inhibitor-like
5	LOC105188195	-1.6495	4.705396	7.43E-10	3.62E-07	insulin (LIRP)
6	LOC105185214	-5.3938	3.30755	3.12E-09	1.32E-06	venom allergen 2-like
7	LOC109504628	-2.5574	4.102739	2.61E-08	9.73E-06	cytochrome P450 6B5-like
8	LOC112588090	-4.8223	1.314016	3.40E-08	1.23E-05	protein ALP1-like
9	LOC105182903	-6.4494	2.735149	3.88E-08	1.33E-05	nose resistant to fluoxetine protein 6
10	LOC105182578	-4.9381	5.886863	1.35E-07	4.12E-05	cytochrome P450 4g15

B)

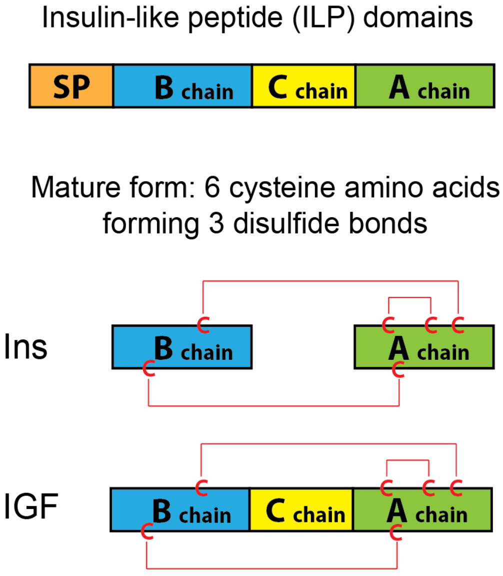

C)

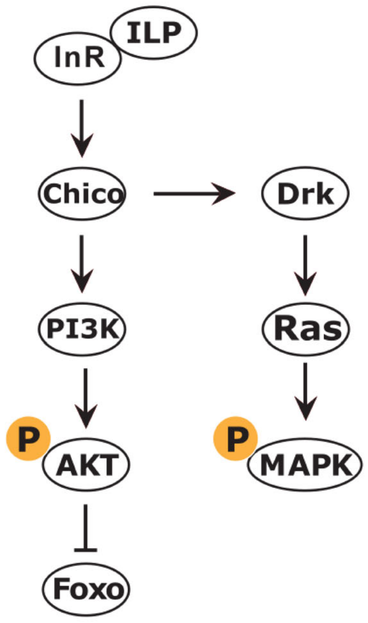

D)

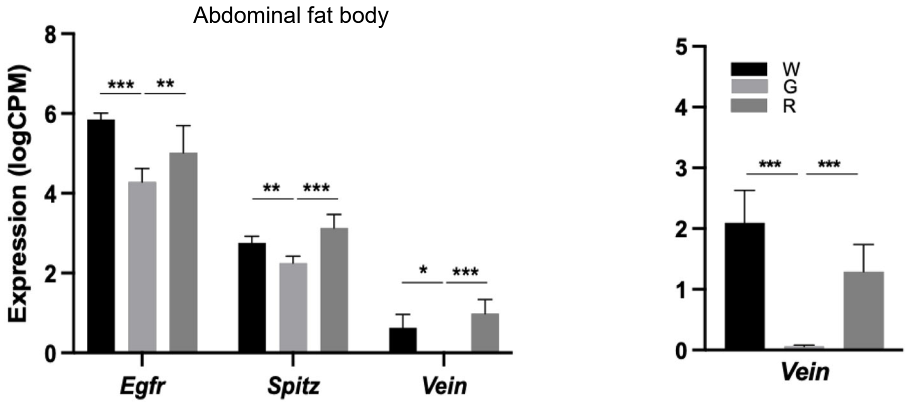

Fig. S3 (**A**) A list of the top 10 characterized gamergate-biased DEGs in the brain compared to workers is sorted by FDR values. Ins (insulin or LIRP) is ranked 5th among the characterized gamergate-biased DEGs in the brains. (**B**) Domain structures in insulin-like peptides (ILPs) and the mature forms of Ins and IGF proteins. Red lines show disulfide bonds. SP: signal peptide. C: cysteine. (**C**) The highly conserved insulin/IGF signaling (IIS) pathway and its downstream targets, AKT and MAPK. AKT and MAPK can also be activated by IIS-independent factors. P: phosphorylation. (**D**) RNA abundance of DEGs in the abdominal fat body and ovary in workers (W, black), gamergates (G, gray) and revertants (R, dark gray). *Egfr: epidermal growth factor receptor*. LogCPM: log counts per million. Data are from four biological replicates per caste. p values from EdgeR are indicated. Bars and error bars represent mean ± SEM. False Discovery Rate (FDR) cut off < 0.05.

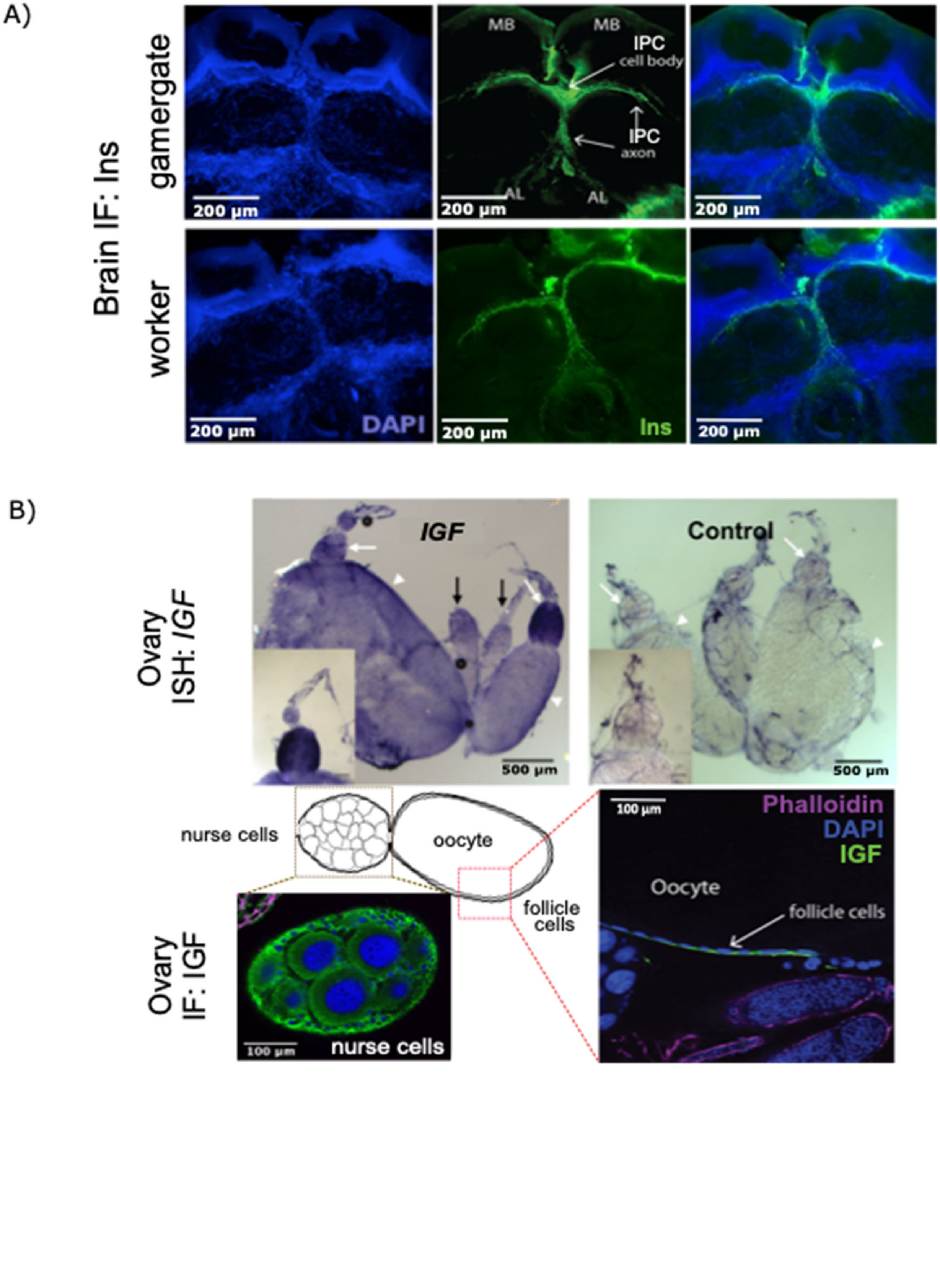

A)
Brain IF: Ins
gamergate
worker
MB
MB
IPC
cell body
IPC
axon
AL
AL
200 µm
DAPI
Ins
B)
Ovary
ISH: IGF
IGF
Control
500 µm
nurse cells
oocyte
follicle cells
Ovary
IF: IGF
100 µm
nurse cells
Phalloidin
DAPI
IGF
Oocyte
follicle cells

Fig. S4. Localizations of Ins and IGF in the brain and in the ovary, respectively. (**A**) immunofluorescence (IF) staining of brain insulin-like peptide (Ins) in a gamergate (upper panel) and a worker (lower panel). Ins protein (green) is localized in the insulin producing cells (IPCs) situated between two mushroom bodies (MBs), and along the axons in both brains of worker and gamergate. Blue represents DAPI. (**B**) Colorimetric *in situ* hybridization (ISH) and IF staining of ovarian IGF. Upper panel, left: localization of *IGF* mRNA by ISH as indicated by arrows; right: a control probe. Lower panel: the nurse cell and the developing oocyte in an egg chamber (EC, left panel) and localization of IGF protein in the EC (right and lower panels). Ovarian IGF protein (green) is localized in the cytoplasm of follicle cells (right panel) and nurse cells (lower-left panel). DAPI stains for DNA (blue). Phalloidin is represented by magenta.

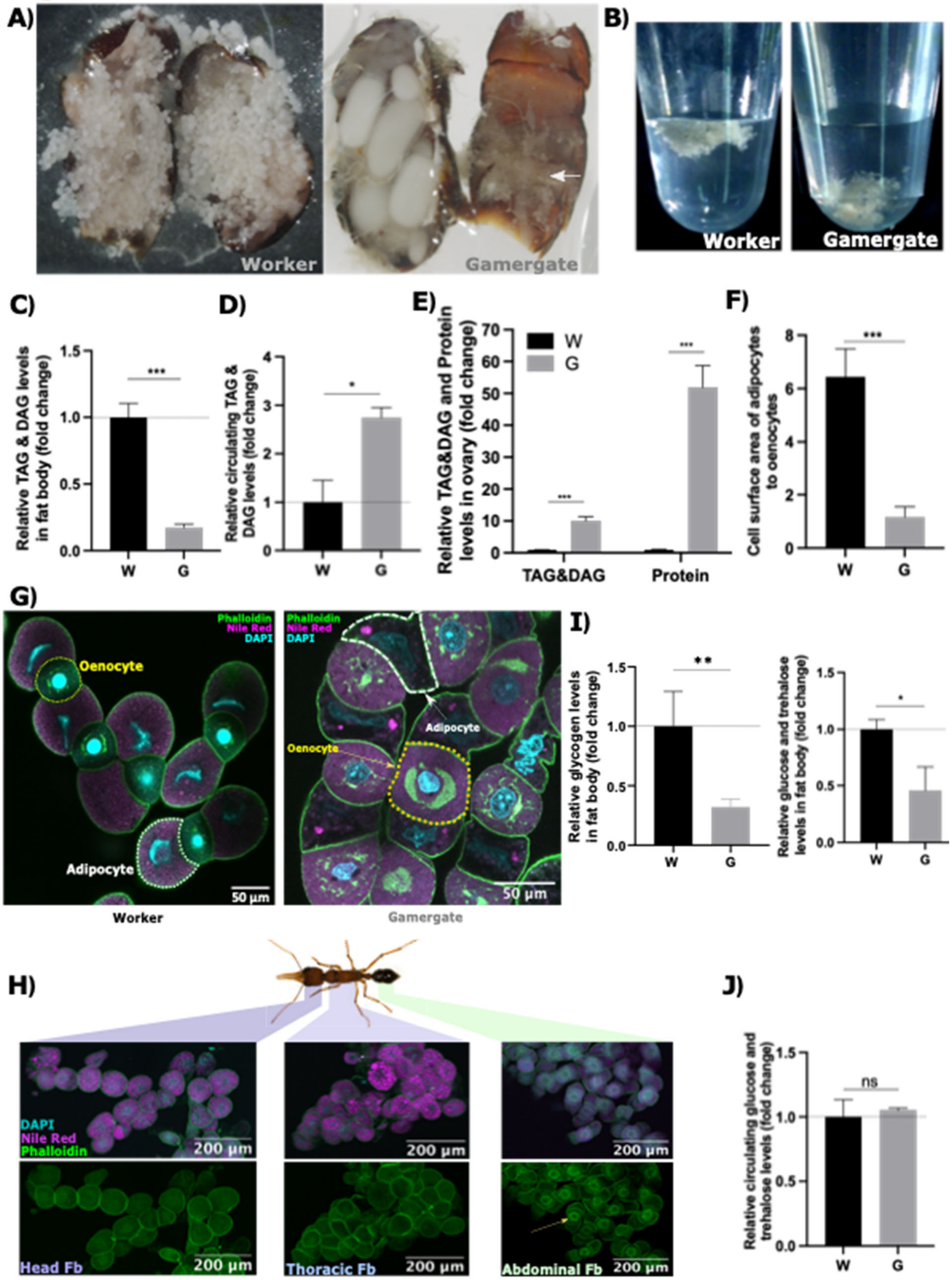
A)
Worker
Gamergate
B)
Worker
Gamergate
C)
Relative TAG & DAG levels in fat body (fold change)
W
G
D)
Relative circulating TAG & DAG levels (fold change)
E)
Relative TAG&DAG and Protein levels in ovary (fold change)
TAG&DAG
Protein
F)
Cell surface area of adipocytes to oenocytes
G)
Phalloidin
Nile Red
DAPI
Oenocyte
Adipocyte
50 µm
Worker
Gamergate
I)
Relative glycogen levels in fat body (fold change)
Relative glucose and trehalose levels in fat body (fold change)
H)
DAPI
Nile Red
Phalloidin
200 µm
Head Fb
Thoracic Fb
Abdominal Fb
J)
Relative circulating glucose and trehalose levels (fold change)
ns

Fig. S5 Lipid depletion in the abdominal fat body during the worker-to-gamergate transition. (**A-B**) Morphological comparisons of abdominal fat bodies in worker *vs.* gamergate. (**A)** Dissected abdomens of workers (W, left) and gamergates (G, right) were imaged by a bright field microscope. Arrow in G indicates the fat body. (**B**) The dissected abdominal fat bodies from workers (left) and gamergates (right) float and sink in saline buffer, respectively. (**C-E**) Comparison of the triglyceride (or triacylglycerol, TAG) and diglyceride (or diacylglycerol, DAG) levels in the fat body (C), hemolymph (D) and ovary (E) of worker and gamergate. (**C**) Quantification of total amounts of TAG/DAG in abdominal fat body per individual worker (W) *vs.* gamergate (G) (n=5 individuals, p<0.001***). (**D**) Quantification of circulating TAG/DAG levels per volume (μl) in worker *vs.* gamergate hemolymph (n=3 individuals, p<0.05*). (**E)** Quantification of TAG/DAG and protein in the ovary: increased lipid and protein levels in individual gamergates (n=8 individuals) compared to workers (n=10 individuals, p<0.001***) may suggest their uptake from hemolymph to ovary for constant oogenesis in gamergates. (**F-H**) Immunofluorescence (IF) staining of the fat body with DAPI, Nile Red and fluorophore-conjugated Phalloidin antibody (cyan, magenta and green, respectively). (**F**) Quantification of the ratio of adipocyte to oenocyte cell surface area in workers *vs.* gamergates (n=3 individuals, p<0.001***). p values are from unpaired t test. Bars and error bars represent mean ± SEM. (**G**) Two fat body cell types: adipocytes (white borderline) and oenocytes (yellow borderlines), are indicated in the abdominal fat body of both worker (left) and gamergate (right). (**H**) Oenocytes (or hepatocyte-like cells), recognized by a green ring structure around the nucleus (stained with Phalloidin, indicated by an arrow in the right lower panel), are found exclusively in the abdominal fat body (Fb), and not in the head and thoracic fat body. (**I-J**) Comparison of the carbohydrate levels in worker *vs.* gamergate. (**I**) Quantifications of levels of glycogen (left) and of trehalose/glucose (right) in the abdominal fat body of each worker *vs.* gamergate (n=5 individuals, p<0.05*). (**J**) Quantification of circulating sugar levels (per microliter) in the hemolymph of worker *vs.* gamergate (n=3 individuals, p=0.70). p values are from unpaired t test. Bars and error bars represent mean ± SEM.

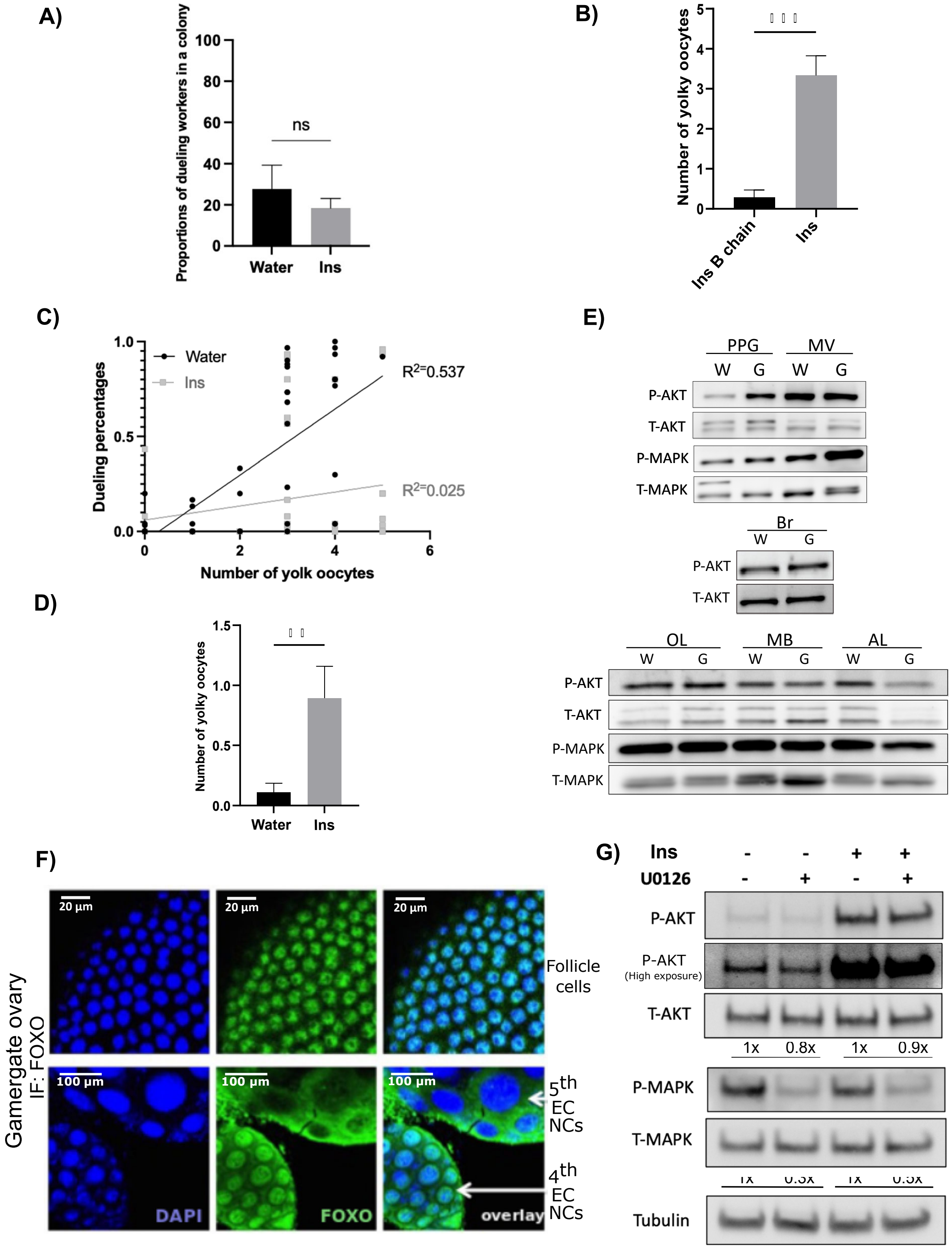
A)
Proportions of dueling workers in a colony
100
80
60
40
20
0
ns
Water
Ins
B)
Number of yolky oocytes
5
4
3
2
1
0
Ins B chain
Ins
C)
Dueling percentages
1.0
0.5
0.0
Water
Ins
R2=0.537
R2=0.025
0
2
4
6
Number of yolk oocytes
D)
Number of yolky oocytes
1.5
1.0
0.5
0.0
Water
Ins
E)
PPG
MV
W G W G
P-AKT
T-AKT
P-MAPK
T-MAPK
Br
W G
P-AKT
T-AKT
OL
MB
AL
W G W G W G
P-AKT
T-AKT
P-MAPK
T-MAPK
F)
Gamergate ovary
IF: FOXO
20 µm
20 µm
20 µm
Follicle cells
100 µm
100 µm
100 µm
5th EC NCs
4th EC NCs
DAPI
FOXO
overlay
G)
Ins - - + +
U0126 - + - +
P-AKT
P-AKT (High exposure)
T-AKT
1x 0.8x 1x 0.9x
P-MAPK
T-MAPK
Tubulin

Fig. S6. Effects of Ins and U0126 on antennal dueling in the workers during transition. (**A-D**) A single injection of Ins with water or Ins B chain as a control into worker abdomen. (**A**) The proportion of dueling *vs.* non-dueling workers in a colony are not significantly affected by Ins injection. (**B**) The number of yolky oocytes in Ins B chain- *vs.* Ins-injected ants. (**C**) Positive correlation between dueling activity and ovary development (the number of yolky oocytes) in control workers (black, R^2=0.5), as opposed to Ins-injected workers (gray, R^2=0.02) that exhibit no positive correlation. (**D**) The number of yolky oocytes of workers in a mature colony with reproductives, injected with water or Ins, were estimated 5 days post injection (n=3 colonies, 60 individuals per condition, p<0.01**). p values are from Mann-Whitney test. Bars and error bars represent mean ± SEM. (**E**) Western blot analysis comparing P-AKT, T-AKT, P-MAPK and T-MAPK levels in the following tissues from worker (W) *vs.* gamergate (G): the postpharyngeal gland (PPG), malpighian vesicle (MV), the whole brain (Br), optic lobe (OL), mushroom body (MB) and antennal lobe (AL). (**F**) IF staining of a transcription factor FOXO in the gamergate ovary detected by *H. saltator* FOXO antibody (green). Nuclear-localized FOXO in the ovarian follicle cells and in the nurse cells (NCs) of the 5th egg chamber (EC) are shown in upper and lower panels, respectively. FOXO localization in the cytoplasm is shown in the 4th EC NCs (lower panels). Nuclei were identified by DAPI (blue). (**G**) Western blot analysis of P-AKT (at low and high exposures), T-AKT, P-MAPK, T-MAPK and tubulin in worker fat body treated with the synthetic Ins peptide and/or U0126. Fold changes (X) of U0126-treated samples are indicated in comparison to either control or Ins-treated samples.

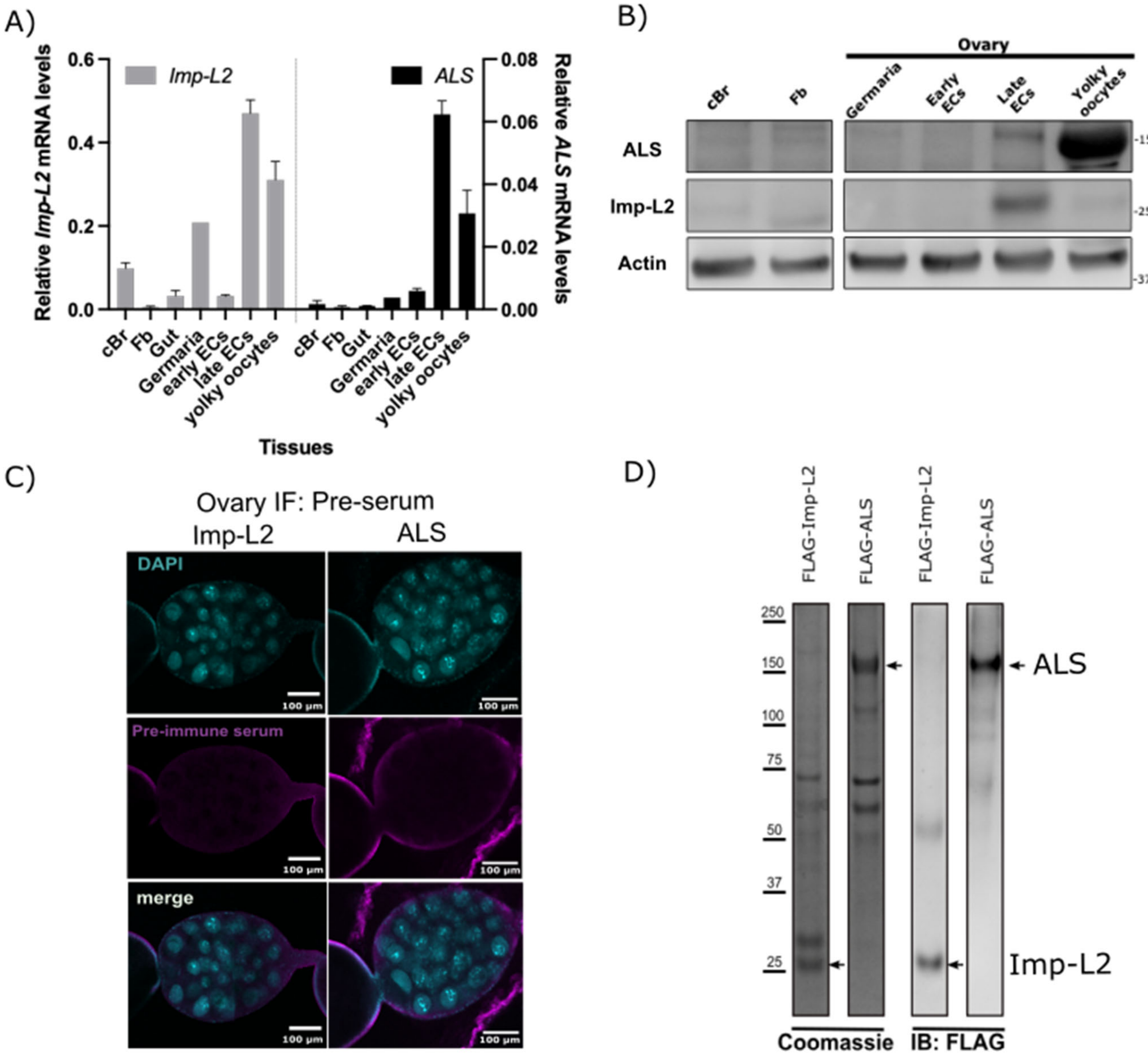
A)
Imp-L2
ALS
Relative Imp-L2 mRNA levels
Relative ALS mRNA levels
0.6
0.4
0.2
0.0
0.08
0.06
0.04
0.02
0.00
cBr
Fb
Gut
Germaria
early ECs
late ECs
yolky oocytes
Tissues
B)
Ovary
cBr
Fb
Germaria
Early ECs
Late ECs
Yolky oocytes
ALS
Imp-L2
Actin
-150kD
-25kD
-37kD
C)
Ovary IF: Pre-serum
Imp-L2
ALS
DAPI
Pre-immune serum
merge
100 µm
D)
FLAG-Imp-L2
FLAG-ALS
250
150
100
75
50
37
25
ALS
Imp-L2
Coomassie
IB: FLAG

Fig. S7. Localization of FOXO, Imp-L2 and ALS in gamergate ovaries. (**A**) RT-qPCR analysis of *ALS* and *Imp-L2* mRNAs in different tissues, such as the central brain without optic lobes (cBr), fat body (Fb), gut and different parts of the ovary from gamergates, including germaria, early egg chambers (ECs), late ECs and yolky oocytes (n=3 individuals). Germaria from three individuals were pooled together. Expression levels were normalized by *ribosomal protein L32 (Rpl32)* gene. Bars and error bars represent mean ± SEM. (**B**) Western blot analysis of Imp-L2 and ALS proteins from different tissues, as indicated. Actin serves as a loading control. (**C**) IF staining of *H. saltator* Imp-L2 and ALS pre-sera in the gamergate ovary (magenta). (**D**) Purified FLAG-tagged Imp-L2 and ALS proteins from insect Sf9 cells were separated by SDS-PAGE and stained with Coomassie Blue (left) and immunoblotted with FLAG antibody (right).

Table S1. Lists of differentially expressed genes (DEGs) identified from the central brain (cBr) without the optic lobes, the abdominal fat body (Fb) and the ovary (Ov) between worker (W) *vs.* gamergate (G) and revertant (R) *vs.* G. Gamergate expression levels were used as a reference for both comparisons. G-biased DEGs, which are up-regulated DEGs in gamergate tissues compared to worker or revertant tissues, are shown in green, while W-/R-biased DEGs are shown in red.
See the Excel file "Table_S1.xlsx"

Table S2. Lists of the predicted biological process (BP) gene ontology (GO) for each gene and the enriched GO terms associated with identified DEGs in different tissues of worker (W), gamergate (G) and revertant (R). The enriched GO terms which are up-regulated in G are shown in green, while W- or R-biased GO terms are shown in red. The enriched GO terms related to fat metabolism and the IIS pathway in fat body (Fb) and ovary (Ov) are highlighted in yellow. p values from Fisher's exact test are indicated. Only terms with significant p values (p< 0.01) are shown.
See the Excel file "Table_S2.xlsx"

Table S3. A list of protein sequences of the 71 Hymenopteran genomes with annotations available in the NCBI RefSeq database.
See the Excel file "Table_S3.xlsx"

Gamergate-biased genes/Worker-biased genes

Used in this study		Hsal gene ID version 8.5	NCBI Reference Sequence (Accession number.Version)	Gene ID	Closest homolog			Tissue sources
Gene symbol	Gene description				*Apis mellifera*	*Drosophila*	*Homo sapiens*	
Ins	Insulin or LIRP	HSALG004952.1	XM_011149533.3	LOC105188195	*AmILP-2*	*Dilp2, Dilp3, Dilp5*	*Ins*	Brain
Crz	corazonin	HSALG005445.2	XM_011155750.2	LOC105191954	*Crz*	*Crz*		Brain
InR1	Insulin receptor 1	HSALG011112.2	XM_025302516.1	LOC105185958	*AmInR2* (Ref. 66)	*InR* (Ref. 66)	*INSR/IGF1R* (Ref. 67)	Abdominal fat body
InR2	Insulin receptor 2	HSALG009512.2	XM_019841795.2	LOC105183944	*AmInR1* (Ref. 66)		*INSR/IGF1R* (Ref. 67)	Abdominal fat body, Ovary
Vg	vitellogenin	HSALG009096.2	XM_011138688.2	LOC105181726	*Vg*			Abdominal fat body
IGF	insulin-like growth factor I	HSALG003795.2	XM_011147513.3	LOC105186969	*AmILP-1*	*Dilp6*	*IGF1*	Ovary
ALS	Acid-labile subunit, convoluted, IGF binding protein (IGFBP), protein artichoke	HSALG003656.2	XM_011151816.3	LOC105189593	*ALS*	*convoluted or ALS*	*ALS*	Ovary
Imp-L2	Imaginal morphogenesis protein-Late 2, Ecdysone-inducible gene L2	HSALG000642.2	XM_011141551.3	LOC105183446	*Imp-L2*	*Imp-L2*	*IGFBP7* (Ref. 63)	Ovary
Hpd-1	4-hydroxyphenylpyruvate dioxygenase	HSALG007876.2	XM_011141398.3	LOC105183352	*Hpd1*	*Hpd*	*Hpd*	Abdominal fat body
Far	fatty acyl-CoA reductase CG5065	HSALG010569.2	XM_011156821.3	LOC105192591	*Far1*	*Far CG5065*	*Far1*	Abdominal fat body
Elovl	fatty acid elongase AAEL008004, CG31522	HSALG002373.2	XM_011153792.3	LOC105190836	*Elovl AAEL008004*	*Elovl CG31522*	*Elov1*	Abdominal fat body
Scd	acyl-CoA Delta(11) desaturase	HSALG013065.2	XM_011153192.2	LOC105190430	*acyl-CoA Delta(11) desaturase*	*desat1*	*Scd*	Abdominal fat body
Fasn	fatty acid synthase-like	HSALG002054.2	XM_025305970.1	LOC112590176	*Fasn*	*Fasn3*	*Fasn*	Abdominal fat body
Egfr	Epidermal Growth Factor Receptor	HSALG001375.2	XM_011155066.3	LOC105191569	*Egfr*	*Egfr*	*erbB-4, Egfr*	Abdominal fat body
Vein	EGFR ligand	HSALG004141.2	XM_025297750.1	LOC105181399	*RIP3*	*Vein*	*NRG*	Ovary
Sb	Serine proteinase Stubble	HSALG009890.2	XM_025298289.1	LOC105189898	*Sb*	*Notopleural*	*Matriptase* (Ref. 98)	Ovary

Table S4. Identifier and homology information of *Harpegnathos saltator* genes discussed in the text.

Oligonucleotides	Sequences (5'-3')	Sources
Vg F primer	CACCTTGACACAACTAGATATAC	Ref. 40
Vg R primer	GTCGGAAGCCTTGATTTG	Ref. 40
Rpl32 F primer	CGTAGGCGATTTAAGGGTCA	Ref. 40
Rpl32 R primer	TTTCGGAAGCCAGTTGGTAG	Ref. 40
ALS F primer	AAACTCCGTCTTGAAGGTACTG	
ALS R primer	GCTCAGATACAATCGTCTGAGG	
Imp-L2 F primer	GGGTAACAATGACCTGCCTATG	
Imp-L2 R primer	TCGACACTGACACGCAATATC	
IGF ISH F probe	GAGAAGCAGGATGAATCG	
IGF ISH R probe	AAATAGAGCTCCAATGTTTTTC	

Table S5. Oligo sequences and their sources (right column) used in this study.

References and Notes

1. S. C. Johnson, P. S. Rabinovitch, M. Kaeberlein, mTOR is a key modulator of ageing and age-related disease. *Nature* **493**, 338–345 (2013).

2. L. Fontana, L. Partridge, V. D. Longo, Extending healthy life span—From yeast to humans. *Science* **328**, 321–326 (2010).

3. M. Tatar, A. Bartke, A. Antebi, The endocrine regulation of aging by insulin-like signals. *Science* **299**, 1346–1351 (2003).

4. M. Tatar, The plate half-full: Status of research on the mechanisms of dietary restriction in Drosophila melanogaster. *Exp. Gerontol.* **46**, 363–368 (2011).

5. R. G. J. Westendorp, T. B. L. Kirkwood, Human longevity at the cost of reproductive success. *Nature* **396**, 743–746 (1998).

6. L. Partridge, N. Alic, I. Bjedov, M. D. Piper, Ageing in *Drosophila*: The role of the insulin/Igf and TOR signalling network. *Exp. Gerontol.* **46**, 376–381 (2011).

7. M. Tatar, Reproductive aging in invertebrate genetic models. *Ann. N.Y. Acad. Sci.* **1204**, 149–155 (2010).

8. E. L. Arrese, J. L. Soulages, Insect fat body: Energy, metabolism, and regulation. *Annu. Rev. Entomol.* **55**, 207–225 (2010).

9. L. Keller, M. Genoud, Extraordinary lifespans in ants: A test of evolutionary theories of ageing. *Nature* **389**, 958–960 (1997).

10. M. Ghaninia, K. Haight, S. L. Berger, D. Reinberg, L. J. Zwiebel, A. Ray, J. Liebig, Chemosensory sensitivity reflects reproductive status in the ant Harpegnathos saltator. *Sci. Rep.* **7**, 3732 (2017).

11. B. Hölldobler, E. O. Wilson, *The Ants* (Belknap Press, 1990).

12. S. A. Ament, M. Corona, H. S. Pollock, G. E. Robinson, Insulin signaling is involved in the regulation of worker division of labor in honey bee colonies. *Proc. Natl. Acad. Sci. U.S.A.* **105**, 4226–4231 (2008).

13. C. Opachaloemphan, H. Yan, A. Leibholz, C. Desplan, D. Reinberg, Recent advances in behavioral (epi)genetics in eusocial insects. *Annu. Rev. Genet.* **52**, 489–510 (2018).

14. C. Opachaloemphan, G. Mancini, N. Konstantinides, A. Parikh, J. Mlejnek, H. Yan, D. Reinberg, C. Desplan, Early behavioral and molecular events leading to caste switching in the ant *Harpegnathos*. *Genes Dev.* **35**, 410–424 (2021).

15. C. A. Penick, M. Ghaninia, K. L. Haight, C. Opachaloemphan, H. Yan, D. Reinberg, J. Liebig, Reversible plasticity in brain size, behaviour and physiology characterizes caste transitions in a socially flexible ant (*Harpegnathos saltator*). *Proc. Biol. Sci.* **288**, 20210141 (2021).

16. J. Gospocic, E. J. Shields, K. M. Glastad, Y. Lin, C. A. Penick, H. Yan, A. S. Mikheyev, T. A. Linksvayer, B. A. Garcia, S. L. Berger, J. Liebig, D. Reinberg, R. Bonasio, The neuropeptide corazonin controls social behavior and caste identity in ants. *Cell* **170**, 748–759.e12 (2017).

17. W. Brogiolo, H. Stocker, T. Ikeya, F. Rintelen, R. Fernandez, E. Hafen, An evolutionarily conserved function of the *Drosophila* insulin receptor and insulin-like peptides in growth control. *Curr. Biol.* **11**, 213–221 (2001).

18. J. Liebig, C. Peeters, N. J. Oldham, C. Markstädter, B. Hölldobler, Are variations in cuticular hydrocarbons of queens and workers a reliable signal of fertility in the ant *Harpegnathos saltator? Proc. Natl. Acad. Sci. U.S.A.* **97**, 4124–4131 (2000).

19. H. Yan, J. Liebig, Genetic basis of chemical communication in eusocial insects. *Genes Dev.* **35**, 470–482 (2021).

20. V. Chandra, I. Fetter-Pruneda, P. R. Oxley, A. L. Ritger, S. K. McKenzie, R. Libbrecht, D. J. C. Kronauer, Social regulation of insulin signaling and the evolution of eusociality in ants. *Science* **361**, 398–402 (2018).

21. C. Slack, N. Alic, A. Foley, M. Cabecinha, M. P. Hoddinott, L. Partridge, The Ras-Erk-ETS-signaling pathway is a drug target for longevity. *Cell* **162**, 72–83 (2015).

22. P. Decio, A. S. Vieira, N. B. Dias, M. S. Palma, O. C. Bueno, The postpharyngeal gland: Specialized organ for lipid nutrition in leaf-cutting ants. *PLOS ONE* **11**, e0154891 (2016).

23. K. E. Brown, M. Kerr, M. Freeman, The EGFR ligands Spitz and Keren act cooperatively in the *Drosophila* eye. *Dev. Biol.* **307**, 105–113 (2007).

24. A. E. Webb, A. Brunet, FOXO transcription factors: Key regulators of cellular quality control. *Trends Biochem. Sci.* **39**, 159–169 (2014).

25. S. S. Lee, S. Kennedy, A. C. Tolonen, G. Ruvkun, DAF-16 target genes that control *C. elegans* life-span and metabolism. *Science* **300**, 644–647 (2003).

26. C. A. Penick, C. S. Brent, K. Dolezal, J. Liebig, Neurohormonal changes associated with ritualized combat and the formation of a reproductive hierarchy in the ant Harpegnathos saltator. *J. Exp. Biol.* **217**, 1496–1503 (2014).

27. H. J. Hsu, D. Drummond-Barbosa, Insulin levels control female germline stem cell maintenance via the niche in *Drosophila*. *Proc. Natl. Acad. Sci. U.S.A.* **106**, 1117–1121 (2009).

28. N. Arquier, C. Géminard, M. Bourouis, G. Jarretou, B. Honegger, A. Paix, P. Léopold, Drosophila ALS regulates growth and metabolism through functional interaction with insulin-like peptides. *Cell Metab.* **7**, 333–338 (2008).

29. L. V. Hun, S. Luckhart, M. A. Riehle, Increased Akt signaling in the fat body of Anopheles stephensi extends lifespan and increases lifetime fecundity through modulation of insulin-like peptides. *J. Insect Physiol.* **118**, 103932 (2019).

30. Y. Yamanaka, E. M. Wilson, R. G. Rosenfeld, Y. Oh, Inhibition of insulin receptor activation by insulin-like growth factor binding proteins. *J. Biol. Chem.* **272**, 30729–30734 (1997).

31. I. Ueki, G. T. Ooi, M. L. Tremblay, K. R. Hurst, L. A. Bach, Y. R. Boisclair, Inactivation of the acid labile subunit gene in mice results in mild retardation of postnatal growth despite profound disruptions in the circulating insulin-like growth factor system. *Proc. Natl. Acad. Sci. U.S.A.* **97**, 6868–6873 (2000).

32. E. Chin, J. Zhou, J. Dai, R. C. Baxter, C. A. Bondy, Cellular localization and regulation of gene expression for components of the insulin-like growth factor ternary binding protein complex. *Endocrinology* **134**, 2498–2504 (1994).

33. L. Manning, J. Sheth, S. Bridges, A. Saadin, K. Odinammadu, D. Andrew, S. Spencer, D. Montell, M. Starz-Gaiano, A hormonal cue promotes timely follicle cell migration by modulating transcription profiles. *Mech. Dev.* **148**, 56–68 (2017).

34. V. Evdokimova, C. E. Tognon, T. Benatar, W. Yang, K. Krutikov, M. Pollak, P. H. B. Sorensen, A. Seth, IGFBP7 binds to the IGF-1 receptor and blocks its activation by insulin-like growth factors. *Sci. Signal.* **5**, ra92 (2012).

35. J. Liebig, H.-J. Poethke, Queen lifespan and colony longevity in the ant *Harpegnathos saltator*. *Ecol. Entomol.* **29**, 203–207 (2004).

36. C. Peeters, J. Liebig, B. Hölldobler, Sexual reproduction by both queens and workers in the ponerine ant *Harpegnathos saltator*. *Insectes Soc.* **47**, 325–332 (2000).

37. S. Séité, M. C. Harrison, D. Sillam-Dussès, R. Lupoli, T. J. M. Van Dooren, A. Robert, L. A. Poissonnier, A. Lemainque, D. Renault, S. Acket, M. Andrieu, J. Viscarra, H. S. Sul, Z. W. de Beer, E. Bornberg-Bauer, M. Vasseur-Cognet, Lifespan prolonging mechanisms and insulin upregulation without fat accumulation in long-lived reproductives of a higher termite. *Commun. Biol.* **5**, 44 (2022).

38. A. R. Armstrong, D. Drummond-Barbosa, Insulin signaling acts in adult adipocytes via GSK-3β and independently of FOXO to control *Drosophila* female germline stem cell numbers. *Dev. Biol.* **440**, 31–39 (2018).

39. A. A. Parkhitko, D. Ramesh, L. Wang, D. Leshchiner, E. Filine, R. Binari, A. L. Olsen, J. M. Asara, V. Cracan, J. D. Rabinowitz, A. Brockmann, N. Perrimon, Downregulation of the tyrosine degradation pathway extends *Drosophila* lifespan. *eLife* **9**, e58053 (2020).

40. M. A. Negroni, E. Jongepier, B. Feldmeyer, B. H. Kramer, S. Foitzik, Life history evolution in social insects: A female perspective. *Curr. Opin. Insect Sci.* **16**, 51–57 (2016).

41. E. J. Shields, M. Sorida, L. Sheng, B. Sieriebriennikov, L. Ding, R. Bonasio, Genome annotation with long RNA reads reveals new patterns of gene expression and improves single-cell analyses in an ant brain. *BMC Biol.* **19**, 254 (2021).

42. A. L. Toth, G. E. Robinson, Worker nutrition and division of labour in honeybees. *Anim. Behav.* **69**, 427–435 (2005).
